# MINFLUX dissects nucleosome and compacting chromatin structures in living cells

**DOI:** 10.1093/nsr/nwaf451

**Published:** 2025-10-21

**Authors:** Hong Xie, Yi Hu, Ruixi Cheng, Xiaohui He, Kaiyuan Liu, Shaoqian Zhang, Bin Xie, He Fang, Yan Yang, Lei Xu, Xi Wang, Jian Lin, Guohong Li, Ji-Song Guan, Hanhui Ma, Min Gu

**Affiliations:** School of Artificial Intelligence Science and Technology, University of Shanghai for Science and Technology, Shanghai 2000093, China; Institute of Photonic Chips, University of Shanghai for Science and Technology, Shanghai 2000093, China; School of Artificial Intelligence Science and Technology, University of Shanghai for Science and Technology, Shanghai 2000093, China; Institute of Photonic Chips, University of Shanghai for Science and Technology, Shanghai 2000093, China; School of Artificial Intelligence Science and Technology, University of Shanghai for Science and Technology, Shanghai 2000093, China; Institute of Photonic Chips, University of Shanghai for Science and Technology, Shanghai 2000093, China; School of Life Sciences and Technology, ShanghaiTech University, Shanghai 201210, China; School of Artificial Intelligence Science and Technology, University of Shanghai for Science and Technology, Shanghai 2000093, China; Institute of Photonic Chips, University of Shanghai for Science and Technology, Shanghai 2000093, China; State Key Laboratory of Epigenetic Regulation and Intervention, Chinese Academy of Sciences, Beijing 100101, China; New Cornerstone Science Laboratory. State Key Laboratory of Metabolism and Regulation in Complex Organisms, Frontier Science Center for Immunology and Metabolism, Hubei Key Laboratory of Cell Homeostasis, College of Life Sciences, Taikang Center for Life and Medical Sciences, Wuhan University, Wuhan 430072, China; School of Artificial Intelligence Science and Technology, University of Shanghai for Science and Technology, Shanghai 2000093, China; Institute of Photonic Chips, University of Shanghai for Science and Technology, Shanghai 2000093, China; School of Artificial Intelligence Science and Technology, University of Shanghai for Science and Technology, Shanghai 2000093, China; Institute of Photonic Chips, University of Shanghai for Science and Technology, Shanghai 2000093, China; School of Artificial Intelligence Science and Technology, University of Shanghai for Science and Technology, Shanghai 2000093, China; Institute of Photonic Chips, University of Shanghai for Science and Technology, Shanghai 2000093, China; School of Artificial Intelligence Science and Technology, University of Shanghai for Science and Technology, Shanghai 2000093, China; Institute of Photonic Chips, University of Shanghai for Science and Technology, Shanghai 2000093, China; School of Artificial Intelligence Science and Technology, University of Shanghai for Science and Technology, Shanghai 2000093, China; Institute of Photonic Chips, University of Shanghai for Science and Technology, Shanghai 2000093, China; School of Artificial Intelligence Science and Technology, University of Shanghai for Science and Technology, Shanghai 2000093, China; Institute of Photonic Chips, University of Shanghai for Science and Technology, Shanghai 2000093, China; State Key Laboratory of Epigenetic Regulation and Intervention, Chinese Academy of Sciences, Beijing 100101, China; New Cornerstone Science Laboratory. State Key Laboratory of Metabolism and Regulation in Complex Organisms, Frontier Science Center for Immunology and Metabolism, Hubei Key Laboratory of Cell Homeostasis, College of Life Sciences, Taikang Center for Life and Medical Sciences, Wuhan University, Wuhan 430072, China; School of Life Sciences and Technology, ShanghaiTech University, Shanghai 201210, China; School of Life Sciences and Technology, ShanghaiTech University, Shanghai 201210, China; School of Artificial Intelligence Science and Technology, University of Shanghai for Science and Technology, Shanghai 2000093, China; Institute of Photonic Chips, University of Shanghai for Science and Technology, Shanghai 2000093, China

**Keywords:** MINFLUX nanoscopy, live-cell imaging, chromatin fiber, nucleosome structure, nuclear organization

## Abstract

The chromatin structure is fundamental for genome compaction and gene transcriptional regulation in the nucleus. Although *in vitro* studies suggest a classical model that 11-nm nucleosome polymers fold into 30 nm fibers, such structures have not been observed *in situ*. In contrast, disordered chains or condensed liquid-like chromatin domains are reported in cells by EM studies and by super-resolution fluorescence microscopy. Do condensed chromatin fibers indeed exist in the cell? We identified a fluorescent dye that preferentially binds to AT-rich regions of DNA and blinks spontaneously to allow single probe visualization. Using three-dimensional (3D) MINFLUX localization, which is extremely low in phototoxicity and high-speed, we observed that a subset of DNA molecules assembles into fiber-like structures co-localized with histones. Native chromatin fibers in living cells are detected in the middle of the nucleus with segments that are variable in width. Most of the chromatin fibers are dismissed after Trichostatin A (TSA) treatment. In some cases, DNA localization even reveals the 3D ultrastructure of individual nucleosomes as 5–10 probes wrapping around an 11-nm cylinder in living cells. Therefore, chromatin fibers (∼30 nm) do exist in living cells, at least in AT-rich regions of the genome. The MINFLUX nanoscopy reveals the native chromatin ultrastructure and its folding to achieve structural compaction in the nucleus.

## INTRODUCTION

In the cell, most genomic DNA is packed into histone proteins and forms compact or relaxed chromatin structures. While each nucleosome consists of a 147-base pair (bp) DNA, wrapping around the histone octamer [1], fitting 3.2 gigabase pairs (Gb) of DNA into the nucleus requires further compaction. On the other hand, nuclear chromatin nanostructuring for gene regulation has emerged as a major challenge, where controversial models predict distinct relationships between the DNA density-related accessibility of transcription factor complexes (TFCs) and gene transcription [2]. Nanostructures and domains of chromatin are important to know how the DNA is packaged in the nucleus, especially in living cells. A hierarchical folding model that nucleosome polymers fold into higher-order chromatin structures [3], especially 30 nm chromatin-fiber has been proposed based on *in vitro* reconstituted materials [4,5]. This model of a condensed chromatin domain with fiber-like structure is frequently used as a textbook illustration of repressive regions with a closed chromatin state. However, there have been several studies in which no distinct higher-order structure in the nucleus was observed by electron microscopy (EM) in fixed cells [6] and by super-resolution imaging in living cells [7–9]. Thus, a remaining question is, what is the fine structure in three-dimensional nucleus chromatin? And if the chromatin-fibers do actually exist in living cells.

Visualized *in vitro*, reconstituted DNA-histone polymers in low salt can form ‘beads-on-a-string’ structures, as multiple 11-nm nucleosome particles linked by 2.5-nm DNA threads [10]. However, to observe this structure in the nucleus is not easy. In Cryo-EM tomography, details of fine-structures depend on the phase contrast between atoms of the molecules and vitreous ice. As the contrast of DNA in vitreous ice is poor [11], chromatin identification lacks unambiguous resolution. Chromatin fibers are observed by Cryo-EM [12], especially near the nuclear envelope region [13]. In transmission EM (TEM), staining by heavy-metal materials can increase the contrast, but they either do not react with DNA or require harsh treatments that destroys the chromatin structure [14,15]. Recently, a method utilizing DNA-binding dye that photo-oxidizes diaminobenzidine (DAB) to induce polymerization, can visualize chromatin structures by EM [6]. As the EM does not directly visualize the DNA dye and spatial variations introduced by DAB, such as polymerization effects and potential diffusion effects of DAB precipitates, the chromatin structures are revealed in an indirect way. To visualize chromatin structures in the nucleus, new efforts are needed to directly localize DNA dye to achieve spatial precision.

To reveal high-order chromatin structures in cells, super-resolution microscopy methods have been developed by visualizing labeled nucleosomes [7,8] and DNAs [9,16] and some chromatin structures are revealed by Hi-C sequencing technologies [17,18]. Under light microscopy with spatial resolution ∼62 nm, condensed chromatin domain ‘blobs’ are detected [7]. Interestingly, single-nucleosome tracking reveals the high-speed motion of chromatin (40–80 nm/50 ms) in living cells, resulting in a liquid-like domain model [19]. Due to the limited spatial resolution and motion blur, it is difficult to detect any fiber-like chromatin structures in the previous studies. It is noticed that to achieve high spatial resolution, illumination intensity utilized in those studies was quite high (∼50 W/cm^2^ at the sample) [9], which might bring additional noise to the system via heating effects.

**Figure 1.**
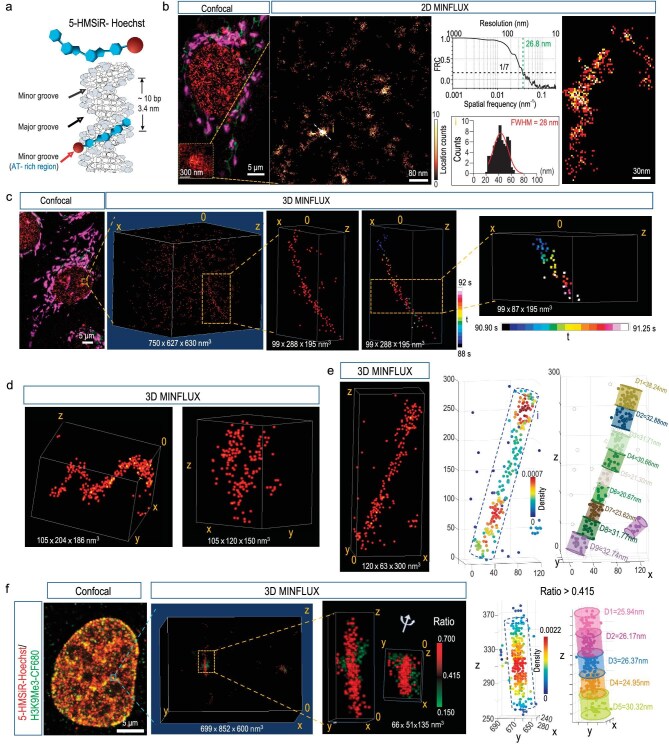
Imaging DNA probe 5-HMSiR-Hoechst illustrates chromatin fibers in living cells and PFA-fixed cells. (a) Illustration shows the binging of 5-HMSiR-Hoechst in minor grooves of DNA. Red, 5-HMSiR. Blue, Hoechst. (b) Confocal image and two-dimensional MINFLUX imaging of DNA stained with 5-HMSiR-Hoechst in living cells showing chromatin fiber-like structures. Magenta (aggregated) and green (monomer) show JC-1 staining for functional mitochondria in living cells. Red shows 5-HMSiR-Hoechst staining for DNA. Middle, FRC curve for the image and FWHM for the detected spot. (c, d) Chromatin fiber-like ultrastructures visualized by three-dimensional (3D) MINFLUX imaging in living cells. DNA is stained with 5-HMSiR-Hoechst (red) in living cells. Magenta (aggregated) and green (monomer) show JC-1 staining in living cells. The fiber-like chromatin structure is imaged within 4 s. Spots are color-coded based on their temporal acquisition sequence (middle). A fraction of the fiber is obtained within 350 ms (right insert). (e) Example of 3D MINFLUX localizations of 5-HMSiR-Hoechst in PFA-fixed cells shows chromatin fiber ∼30 nm within the nucleus. Right, the fiber is divided into 9 segments, diameters of each segment are shown. (f) Chromatin fiber-like ultrastructure visualized together with H3K9me3-CF680 by dual-color 3D MINFLUX imaging. Histone is visualized by directly labeled primary antibodies conjugated to CF680 (anti-H3K9me3, green). DNA is visualized by 5-HMSiR-Hoechst (red). Two signals in dual-color 3D MINFLUX are divided according to signal ratio of each location in two spectral fractions (see Fig. S7 for details). The DNA fiber–related signal locations (ratio >0.415) are divided into 5 segments, diameters of each segment are shown. H3K9me3-CF680, H3K9me3 labeled with primary antibody conjugated with CF680.

We reasoned that MINFLUX [20], a recently introduced microscopy method for localizing fluorophores with a minimal number of detected photons, could greatly improve the study of chromatin structures. MINFLUX localization of a fluorophore is performed iteratively by continually shifting the minimum closer to the fluorophore [21–24], thereby, to achieve spatial precision requires low background noise and blinking ability of the dye [25]. In this study, the illumination intensity in MINFLUX is as low as 23 μW/cm^2^. In MINFLUX, an active feedback position stabilization system, which utilizes back-scattered light from reflective nanorods that are immobilized along with the sample, achieves remarkable stability (Fig. S1), exhibiting standard deviations substantially below 1 nm (x, y and z axis) over a period of hours [22]. Furthermore, localization of each fluorophore was performed ∼93.7 μs in 3D and 29.0 μs in 2D (Fig. S2), which largely minimized the motion-induced blur of chromatin structures. Hoechst 33342, a DNA probe, binds preferentially to AT-rich DNA in the minor groove of double strand DNA (Fig. 1a). A recently developed DNA dye, hydroxymethyl silicon-rhodamine (5-HMSiR-Hoechst), is cell-permeable with extremely low background fluorescence and shows stable ‘blinking’ in cellular pH conditions [26]. Therefore, this DNA probe is used for single molecule localization by MINFLUX in fixed cells with a high precision (FWHM_x_ = 1.08 nm, FWHM_y_ = 0.96 nm, FWHM_z_ = 0.63 nm) (Fig. S3). Here, we report that direct DNA dye localization by MINFLUX pushes the resolution of the chromatin structure to nanometer resolution in 3D space. The high spatial resolution of DNA probe localization allows the direct detection of fine chromatin structures as the chromatin fibers are in the middle region of some nuclei. In a few cases, it even reveals DNA locations that form single nucleosome-like structures. This study provides the evidence of condensed domains as chromatin fibers do indeed exist in living cells.

## RESULTS

### Emergence of 30-nm DNA fiber-like chromatin ultrastructure among MINFLUX locations in living and fixed cells

Upon binding to DNA, 5-HMSiR-Hoechst excites strong florescence signals and shows spontaneous blinking [25], which leads to florescence staining in the nucleus of living human osteosarcoma U2OS cells. Confocal microscopy imaging reveals that 5-HMSiR-Hoechst is cell permeable and stains the nucleus very well in living U2OS cells with intact mitochondrial function as shown by JC-1 staining [27] (Fig. 1b and Fig. S4a). MINFLUX enables direct locating of fluorophores with a precision of ∼2.4 nm in the focal plane and ∼1.9 nm along the optic axis in 3D configuration [22]. For 5-HMSiR-Hoechst, MINFLUX enables location with a precision of ∼1 nm in the focal plane and 0.6 nm along the optic axis (Fig. S3). Interestingly, the spontaneous blinking events of 5-HMSiR-Hoechst allows the MINFLUX localizing of DNA structures from a dense background by sequential visualization at different time points (Fig. S4b). In living cells, the MINFLUX locations are distributed in domains of condensed signals. In 2D MINFLUX localizing, we calculated the Fourier ring correlation (FRC) curve of the localization data (Fig. 1b, middle). The threshold value of 1/7 gave 26.8 nm as a resolution estimate, suggesting the parameter of the chromatin fiber structure. Consequently, we measured some high-density spots which showed peaks with a width of 12 nm to ∼28 nm (Fig. 1b). This observation was consistent with the report that measured chromatin domain FWHM to be ∼31 nm, using the same dye in TIRF SMLM of living human fibroblasts [26]. In a few cases, we could observe some twisted chromatin fibers in the 2D MINFLUX localization (Fig. 1b).

To further validate the 3D structure in detail with higher confidence, we visualized this structure under the 3D MINFLUX localizing configuration (Fig. 1c). Although some chromatin ultrastructures might be moving in living cells, making them impossible to be visualized, we do identify some fiber-like structures in the MINFLUX imaging field within 350 ms (Fig. 1c). Some structures are persistent for at least 3–4 s (Fig. 1c and Fig. S2f). These observations indicate that a small fraction of chromatin fibers in living cells are relatively stable at the scale of seconds, in contrast to the liquid-like domain model [19]. We observed some locations of chromatin forming fiber-like high-density clouds, a few segments of the fibers showed a width of ∼30 nm (Fig. 1d). The existence of 30-nm fiber has been observed in reconstituted *in vitro* systems by X-ray [4,5], but it has never been reported in the nucleus by TEM, Cyro-EM or other florescent imaging systems [6,16]. More detailed structures could be revealed by focusing on the fiber like DNA strings. Three-dimensional MINFLUX locations identified many fiber-like chromatin ultrastructures with different widths (Fig. S5). Interestingly, in some cases, the identified higher-order chromatin domains contain multiple fiber-like chromatin structures (Fig. 1d and Fig. S5b). It was fascinating to obtain the fiber-like chromatin structures in living cells, given the motility of chromatin [8] or even Brownian motion of biomolecules. First, condensed chromatin fibers might reflect a closed chromatin state, where less motilities are presented. In this case, only stable components in the chromatin fibers (at least stable for 3–4 s) are obtained by MINFLUX imaging in living cells. Fractions of moving chromatin domains are not able to be visualized in the living cell. Second, the MINFLUX localization of 5-HMSiR was performed at ∼93.7 μs per localization (Fig. S2c and d), which largely reduced the motion effect. And each localization consisted of 10 iterations of the re-centering process with 50–170 kHz rate (Fig. S2a, b), which excluded fast-moving particles.

Furthermore, to avoid motility of chromatin fibers in order to reveal the ultrastructure more clearly, we used paraformaldehyde (PFA) to fix the cell (Fig. 1e and Fig. S6a). In the fixed cells, we detected that some chromatin fibers (∼30 nm) were connected with DNA chain-like structures (Fig. S6d), which might be subject to fast movement and were never observed in living cells. In the meantime, as expected, MINFLUX localization detected more fluorophore localizations on the chromatin fiber in the fixed cells, ∼2–3-folds higher than that in living cells. In addition, the 30 nm chromatin fiber revealed by 3D MINFLUX localizing is not a uniform structure, it can be further divided into segments with different densities and widths (Fig. 1e and Fig. S6b). Fiber segments with distinct diameters and densities are intermingled with the chromatin fibers. The chromatin fibers stretch up to >500 nm (Fig. S6d). It is noticed that the absolute DNA densities in these chromatin fibers are estimated to be quite high (34 Mbp/μm^3^) (Fig. S6b, c), considering each probe molecule binds to 12 bp of AT-rich DNA and they cover 80% of the region of DNA at most [28]. The densities of these fibers (Fig. S6c, 8.87–69.87 Mbp/μm^3^) are lower than the constitutive heterochromatin of the inactive nuclear compartment [9] (>300 Mbp/μm^3^), suggesting room for regulatory accessibility in chromatin fibers.

Importantly, those detected chromatin fiber structures were closely associated with histones, especially the repressive marker of the closed chromatin state. We imaged histone H2A, a core component of nucleosome, or H3K9me3, a repressive histone marker, using primary antibodies labeled with the fluorophore CF680, together with the DNA dye, 5-HMSiR-Hoechst. With a modified imaging buff system (see Methods for details), we were able to divide DNA signals from histone signals in a dual-color 3D imaging configuration (Fig. S7a–d). Although the size of the antibody is larger than the size of nucleosomes, we can detect some condensed DNA signals in string-like form that are adjacent to histone H2A signals (Fig. S7e), suggesting the ultrastructure of chromatin fibers. Detected chromatin fibers were also co-localized with repressive histone marker H3K9me3 (Fig. 1f and Fig. S7f), further confirming the chromatin fiber (30 nm) as the closed chromatin state.

**Figure 2.**
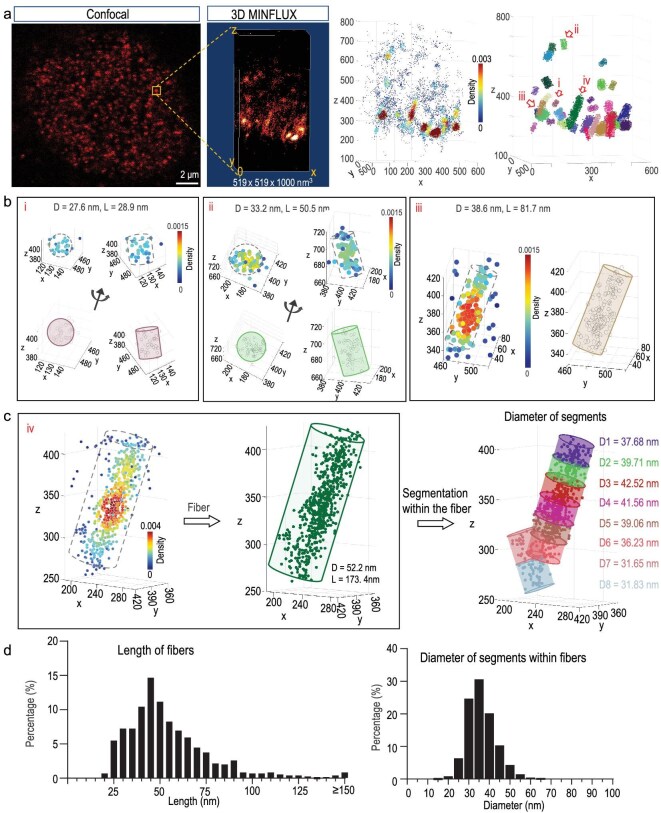
Three-dimensional (3D) MINFLUX imaging reveals chromatin fibers. (a) 3D MINFLUX imaging of DNA stained with 5-HMSiR-Hoechst in PFA-fixed cells showing chromatin fiber-like structures. Right, example of individual chromatin fibers within this area. (b) Examples of chromatin fibers in (a) (i–iii). D, dimeter. L, length. Length is defined as the long-axis of condensed probe locations. (c) Example shows the segmentation analysis of chromatin fiber by fitting with cylinder modals. (d) Statistics showed the length of each observed chromatin fiber (*n* = 687 from 26 regions, two regions per cell). Diameters of each segment with the fibers are shown (*n* = 978 segments in 687 fibers from 26 regions, two regions per cell).

In total, we detected 1085 chromatin fibers from 57 fixed cells. Some fibers are straight, others form some curved structures with different widths (Fig. 2a). To quantify the lengths of these chromatin fibers, we identified each fiber using the Density-Based Spatial Clustering method (DBSCAN2, see Methods for details) and the length of the long axis was determined as the length of the condensed signals (Fig. 2b). To quantify the diameter and length of each chromatin fiber with varied widths, we further divided each condensed signal into separate fibers by fitting with cylinder models (see Methods for details) (Fig. 2c). The total lengths of most detected chromatin fibers are ∼40–75 nm long (Fig. 2d), some of them stretch up to >400 nm (Fig. S6b, d). DNA probes reveal that the chromatin fiber-like structures show a preference of 35 nm in diameter (Fig. 2d and Fig. S8). Interestingly, similar to U2OS cells, we also detected fiber-like DNA structures in cultured neurons (Fig. S9). In contrast to the reported native chromatin fibers, that are only observed near the nuclear envelope by EM [13], most of the chromatin fibers are detected in the middle part of some nuclei in this study. Given that the probe labels AT-rich regions, this observation might only reflect local structural features, but not across the whole genome. Therefore, MINFLUX localizing of 5-HMSiR-Hoechst, the DNA probe, reveals chromatin fiber units as distinct chromatin domains in living cells.

### Single nucleosome-like structures visualized *in situ* by MINFLUX in living cells

**Figure 3.**
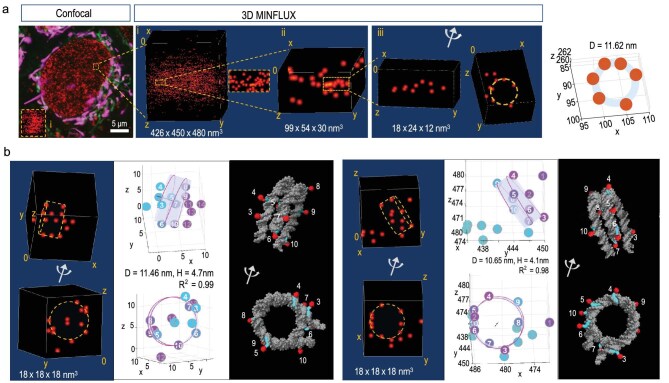
Distributions of DNA probe 5-HMSiR-Hoechst illustrate nucleosome structures in living human U2OS cells. (a) Confocal image and three-dimensional (3D) MINFLUX imaging of DNA stained with 5-HMSiR-Hoechst in living cells. Magenta (aggregated) and green (monomer) show JC-1, staining for functional mitochondrial in living cells. Red shows 5-HMSiR-Hoechst staining for DNA. 3D MINFLUX localizations showing ring-like structure. Right, location data in (iii) fitted by the ring model (diameter = 11.62 nm, *R*^2^ = 0.81). (b) Two representative examples of single nucleosome detected by 3D MINFLUX. Probe locations are fitted by a helical and cylindrical model (middle). White digits indicate probes on the helical structure. Illustrations demonstrate the mapping of probes onto the nucleosome model (PDB 7Y5W) (right).

In a few cases, we even detected single nucleosome-like structures in the MINFLUX localization of DNA dye (Fig. 3a). In a volume of 426 × 450 × 480 nm^3^, many fiber-like chromatin compaction structures are revealed. In 18 × 24 × 12 nm^3^ scale, the probe locations form a ring-like structure, suggesting DNA wrapping around the 11-nm nucleosome (Fig. 3a). We reasoned that as Hoechst binds to the minor groove of the double DNA strand at the AT-rich region, while each molecule binds to 12 bp DNA [28] in 140 bp of nucleosome DNA, we should be able to identify up to 10 molecules fitting to the locations of the nucleosome model (Fig. 3b). Many nucleosome-like structures are observed, with 5–10 probes in each fitting (probably due to the variations of AT-rich regions in nucleosome DNAs, Fig. S10a). Fitting the 3D MINFLUX locations with the ring-like model results in two peaks under 13 nm, one at 11.5 nm and another at 12.5 nm (Fig. S10b). We reasoned that the latter one might be due to the orientation angle of the fitting plate. Thus, the observed mean diameter of the DNA strand around the nucleosome is 11.5 nm, which is consistent with the parameter of nucleosome observed in EM and X-ray. For more general conditions, we fitted the locations to a helical and cylindrical model (details in Methods section). In a particular 18 × 18 × 18 nm^3^ domain, we are able to fit the probe locations around a single nucleosome in 3D space. In this space, among the 14 locations, 8 probes attach to the cylinder, with a diameter of 11.46 nm and 4.7 nm height (Fig. 3b). Further examples show the fitting of probe locations in individual nucleosomes (Fig. 3b and Fig. S11). The low occupancy of DNA probes on the nucleosome makes it difficult to determine the position of linker histone H1 and linker DNAs. In each imaged area, individual nucleosomes and nucleosome polymers are detected. The average widths of individual nucleosomes are 11.55 nm (fixed cell) and 11.61 nm (living cell), consistent with the *in vitro* data (Fig. S12b, c). It is noted that 5–10 probes occupy 60–120 bp DNA, which correlates with ∼43% to 81% AT-rich regions in the nucleosome DNA. Nucleosomes with low AT-rich regions cannot be detected by the model fitting approach. Therefore, not all probe signals could be fitted in nucleosome models.

In some cases, the 3D MINFLUX localizing of DNA probes were fitted into a cylindrical model with 2 nucleosomes (Fig. S13a). In fact, the dimer-like nucleosome units were observed in many loci, where the width of the cylinders are ∼11.5 nm, similar to that of the individual nucleosomes (Fig. S13b). We found that locations in chromatin fibers and some condensed regions could fit in dimer-nucleosome units (Fig. S14). We were able to fit 6 nucleosome dimers into one domain, resulting in a 12-mer chromatin structure within a small domain of chromatin fiber (Fig. S15). Furthermore, as a control experiment, MINFLUX localization was able to identify nucleosome structures on reconstituted nucleosome arrays and reconstituted chromatin fibers (Fig. S16). These observations might suggest nucleosomes are organized within each chromatin fiber.

### TSA disrupts chromatin fiber structures

**Figure 4.**
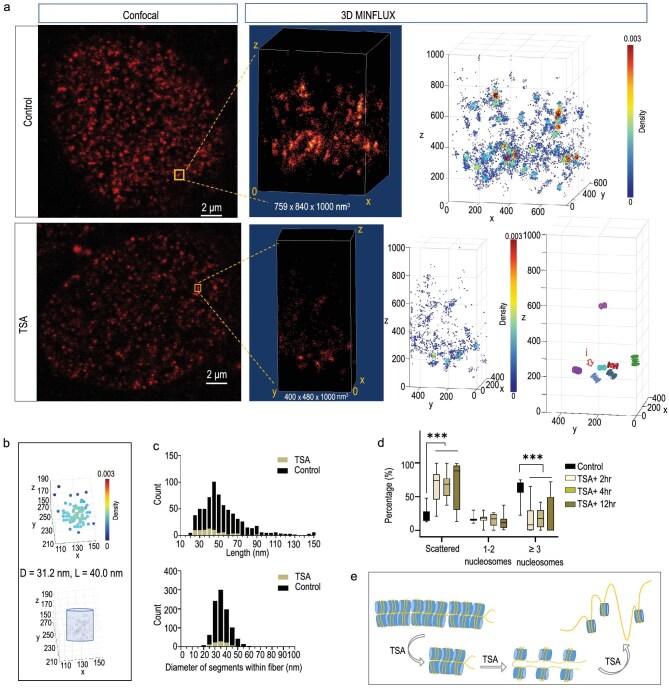
Three-dimensional (3D) MINFLUX imaging reveals TSA-induced alternation of chromatin fibers. (a) Confocal image and 3D MINFLUX imaging of DNA stained with 5-HMSiR-Hoechst in PFA-fixed cell showing one representative example each of TSA-induced cell and control cell. Chromatin fiber claps at 2 h after TSA treatment. Middle, 3D MINFLUX localizations of the DNA probes are color-coded with local density. Right insert, example of remaining chromatin fibers. (b) Examples of probe localizations in chromatin fiber treated by TSA in (a). (c) Statistics show the alternations of chromatin structure according to regional MINFLUX localizing density of DNA probes between the TSA treated sample (*N* = 84 fibers, 99 segments from 37 regions, one region per cell) and control sample (*N* = 687 fibers, 978 segments from 26 regions, one region per cell). (d) TSA treatment alters the accumulation of nucleosome polymers. N_control_ = 12 cells, N_TSA+2hr_ = 9 cells, N_TSA+4hr_ = 13 cells, N_TSA+12hr_ = 15 cells. ANOVA: Scattered: F(3,45) = 10.20, *P* < 0.0001; Dunnett’s multiple comparison test, P_control v.s. TSA+2hr_ = 0.0005, P_control v.s. TSA+4hr_ = 0.0001, P_control v.s. TSA+12hr_ < 0.0001,1–2 nucleosomes: F(3,45) = 1.219, *P* = 0.3139; Dunnett’s multiple comparison test, P_control v.s. TSA+2hr_ = 0.9988, P_control v.s. TSA+4hr_ = 0.9860, P_control v.s. TSA+12hr_ = 0.2820, ≥3 nucleosomes: F(3,45) = 12.62, *P* < 0.0001; Dunnett’s multiple comparison test, P_control v.s. TSA+2hr_  < 0.0001, P_control v.s. TSA+4hr_  < 0.0001, P_control v.s. TSA+12hr_ < 0.0001. ***P < 0.001. In all box plots, whiskers represent the minimum and maximum values, while the box encapsulates the interquartile range, depicting the 25th and 75th percentiles along with the median. (e) Illustration of the TSA induced chromatin structural changes.

To further validate the observed ultrastructure of chromatin fiber by MINFLUX localization, we applied Trichostatin A (TSA) (Fig. 4a), which is a histone deacetylase inhibitor that strongly triggered chromatin remodeling in U2OS cells. TSA induces increased acetylation of histone tails and alters the charges on nucleosomes so that DNA is released from histone and compacted chromatin structures are converted to relaxed structures. At the same time, TSA-treatment is closely related with transcriptional activation of the gene, thereby TSA-induced chromatin remodeling directly influences gene transcriptional regulation. In contrast to the control condition, at 12 h after TSA treatment, MINFLUX localizing of DNA probes suggest large chromatin structural changes in fixed cells (Fig. 4a) and in living cells (Fig. S17). Under this condition, long chromatin fiber structures are hardly observed and scattered in the 3D field (Fig. 4a, b). The 3D MINFLUX localizing data of DNA probes in the TSA-treated data shows significant loss of high-density chromatin fiber-like structures (Fig. 4c). Furthermore, in TSA-treated cells, when the chromatin fibers are detected, the length of those fibers reduced to <50 nm (Fig. 4b). Statistically, the diameters of the clustered localizations in the TSA-treated samples remained the same (∼30 nm) as those in the control condition (Fig. 4c).

The occurrence of chromatin fiber-like structures after TSA treatment reduced to <10% compared to the control condition. In control conditions, 60% of the MINFLUX localizations are in the dense regions, which are fitted by nucleosome polymers of >3 nucleosomes. After TSA treatment, MINFLUX localizations in the dense regions reduced to 20%, and major part of those (>70%) are scattered in many loss structures (Fig. 4d). Therefore, the observation of 30-nm chromatin fibers containing nucleosome polymers are subject to collapse after TSA treatment (Fig. 4e). The nucleosome polymers are subject to disassembly upon acetylation of histones.

## DISCUSSION

This study shows that 3D MINFLUX nanoscopy is a powerful tool for imaging dense structures in the nucleus with nanometer isotropic precision. By localizing florescence DNA probes with blinking features, chromatin fibers are visualized in living cells. Our 3D MINFLUX imaging builds on pioneering advancements in super-resolution chromatin visualization, while directly resolving the chromatin structure using fluorogenic probes (e.g. 5-HMSiR-Hoechst) *in situ*. This approach complements prior methodologies such as PALM [7,8] and dynamic chromatin tracking [9], which elucidated nucleosome mobility in live cells. Crucially, MINFLUX bypasses osmium staining artifacts inherent to ChromEMT and achieves isotropic 1-nm resolution in 3D, enabling direct detection of chromatin fiber geometries unattainable with earlier techniques and photoactivated localization microscopy (PALM).

In addition, when compared to other super-resolution microscopy methods, MINFLUX localization requires a significantly lower illumination intensity (6 magnitudes lower than PALM [7,8]), minimizing potential light-induced distortions. Assigning localization clouds to individual nucleosomes and chromatin fibers simplifies unit counting and provides quantitative interpretations of spatial distributions of DNA in each chromatin domain and their arrangements.

The actual limitation in accessing the exact chromatin ultrastructure within a highly compacted nucleus is when it comes to illuminating the structure. For the EM method, efforts to enhance the signal contrast might include harsh conditions, which might break the ultrastructure of chromatin [15]. Others utilize enzymatic amplification methods, which brought concerns of spatial precision in nanometer domains. Florescent DNA dyes are nanometer in size and increase florescence upon binding to DNA [25], therefore, they are good candidates to reveal the chromatin structure. However, the high density of DNAs compacting in the nucleus is a major challenge when dissecting the chromatin structure using florescence microscopy, due to optic limitations. In this study, the spontaneous blinking character of DNA dye, 5-HMSiR-Hoechst, may have played a critical role in illumining the chromatin fiber. SYTOX Orange is another fluorophore with a blinking event. However, studies using SYTOX in fibroblasts and in HeLa cells did not obtain chromatin fiber structures [9]. Besides the advances in resolution by MINFLUX in this study, the preference of binding site by Hoechst might also count. SYTOX Orange shows a similar affinity to both GC- and AT-rich DNA, in contrast with Hoechst, which binds preferentially to AT-rich DNA [28]. AT-rich regions are typically associated with heterochromatin, while GC-rich sequences are more likely to be found in dispersed euchromatin [29]. AT-rich genes are more likely to be tissue-specific (silenced in most tissues). Therefore, 5-HMSiR-Hoechst might preferentially label the condensed chromatin fibers and increase the signal-to-noise contrast, when compared to SYTOX Orange. On the other hand, 5-HMSiR-Hoechst might limit detection of nucleosomes with low AT contents and its blinking issue could underestimate nucleosome counts in chromatin structures. In addition, as 5-HMSiR-Hoechst binds to DNA and may interact with some enzymes involved in chromatin remodeling, this probe could have influenced the stability of the chromatin structure to observe those fibers.

Due to the combination of reversible blinking and probe exchange, the same probe is hardly observed twice in the same position by 3D MINFLUX localizing, leaving no room for further improvement of position precision by combining multiple localizations. For the same reason, the 3D MINFLUX imaging did not cover the positions of all probes in our study, resulting in a subsampling of the chromatin structure. Therefore, only a few 3D MINFLUX locations of DNA probes are able to be fitted into nucleosomes.

Revealing chromatin structures in living cells is challenging, as previous studies by labeling histone protein indicate a high-level of chromatin dynamics in living cells [7,8]. Chromatin motility reduced the events of probe localization by MINFLUX in living cells more so than in fixed cells. However, MINFLUX localizations are still able to obtain the chromatin fiber-like structure in living cells. This might depend on two factors: components in the condensed chromatin fiber are moving relatively much slower than the relaxed DNA; effective fluorescence detection rate of ∼60 kHz in MINFLUX allowed a complete localization within 93.7 μs so that particles that are moving within nanometer regions could be localized. By these means, the visualization of fiber-like domains in living cells indicates the existence of some relatively stable chromatin fibers, at least in a small fraction of the chromatin. In contrast to chromatin fibers detected near the nuclear envelope by EM [13], most of the fiber-like structures in living cells are presented in the middle of the nucleus by MINFLUX imaging.

In conclusion, we show that 3D MINFLUX nanoscopy enables the 3D mapping of chromatin structures in the nucleus, visualizing the fiber-like structure of chromatin with single-digit nanometer resolution. This study shows that the 30-nm chromatin fiber as heterochromatin does indeed exist in living cells. In conjunction with appropriate probes, MINFLUX nanoscopy thus opens a new field in the analysis of chromatin structures and its remodeling for genome regulation in (living) cells.

## MATERIALS AND METHODS

### Live cell DNA staining with 5-HMSiR-Hoechst probe

U2OS cells (human osteosarcoma cell line) and primary cultures of neurons were prepared as previously reported [30]. Glass-bottom dishes (Thermo Scientific, diameter: 20 mm) were pre-treated before cell culture. The dishes were coated with poly-D-lysine (PDL, Sigma Aldrich) overnight to facilitate cell adhesion. Subsequently, the dishes underwent 1-h incubation with a gold nanoparticle solution (150 nm, BBI Solutions). After each pre-treatment step, the dishes were thoroughly washed with sterilized ddH_2_O to remove excess reagents. U2OS cells were then cultured on the pre-treated glass-bottom dishes. After 12 h of growth, the cells were gently washed three times with prewarmed PBS. Subsequently, fresh maintenance medium was added to the dishes. The staining solution, consisting of 5-HMSiR-Hoechst with a final concentration of 15 nM, was then introduced, and the dish returned to the incubator. The staining duration was set at 2h. The live cell samples were imaged without washing at room temperature.

### MINFLUX nanoscopy

MINFLUX nanoscopy was conducted using an Abberior Instruments MINFLUX microscope equipped with a 1.4 NA,100× oil objective lens, featuring essential components such as a 642-nm continuous-wave excitation laser, a 405-nm continuous-wave activation laser, a spatial light modulator-based beam shaping module, and an electro-optical detector-based MINFLUX scanner [21,^31^]. The microscope was also equipped with two avalanche photodiodes and fluorescence filters (650 to 750 nm) for detecting fluorescence photons emitted from the sample. A confocal module with a 642-nm laser was used for the identification of DNA/chromatin regions within the cell nucleus. Subsequently, these regions were selected as regions of interest (ROIs). Following this selection process, the MINFLUX microscope engaged the MINFLUX642 laser channel while simultaneously choosing either 2D MINFLUX or 3D MINFLUX imaging modes for data acquisition in the cy5 near wavelength range (excitation light within the 650–685 nm range). The MINFLUX stabilization system is implemented to mitigate fluctuations within the imaging system during MINFLUX nanoscopy. Nanoscale gold particles, acting as fiducial markers, are integrated into the imaging setup to enable continuous monitoring and correction of potential drift or instability in the microscope system. Facilitated by this stabilization system, the MINFLUX system exhibits consistent stability in the XYZ directions, maintaining fluctuations within a tight range of 1 nm throughout the entirety of the imaging experiments. This ensures the precision and reliability of the MINFLUX imaging process [21,^31^]. The MINFLUX dataset was imported into MATLAB for localization and structural analysis.

## Supplementary Material

nwaf451_Supplemental_Files

## Data Availability

The MATLAB code used for data analysis of this study is available at: https://github.com/Xiehong-usst/DNA-code..git
