# Supplementary material for: MINFLUX dissects nucleosome and compacting chromatin structures in living cells
Source: Natl Sci Rev. 2025 Oct 21;13(3):nwaf451. doi: 10.1093/nsr/nwaf451 (PMC12875114; doi:10.1093/nsr/nwaf451)
Supplement: nwaf451_Supplemental_Files [file nwaf451_supplemental_files.zip › Supplemental Table.docx]

**Supplemental Table. Summarized fiber parameters in living and PFA-fixed cells.**

|  | **Length of fibers (nm)** | **Diameter of fibers (nm)** | **Number of**  **fibers / µm^3^** |  |
| --- | --- | --- | --- | --- |
| **PFA-fixed U2OS cell** | **55.08±0.97** | **36.21±0.227** | **75.26± 13.83** |  |
| **PFA-fixed U2OS cell**  **(+TSA)** | **46.95±2.32** | **35.07±0.62** | **15.65± 3.78** |  |
| **Living U2OS cell** | **48.99±1.84** | **29.69±0.99** | **NA*** |  |
| **PFA-fixed primary neuron** | **45.59±0.69** | **31.54±0.22** | **73.88± 28.69** |  |

NA* Due to motility of chromatin fibers in living cells, detection of chromatin fibers in living cells is subsampling from the fixed ones, resulting in significant reduction of detectable chromatin fibers, compared to the fixed cells. To avoid misleading message to compare the parameters, we did not include data in this table.
