# Supplementary material for: MINFLUX dissects nucleosome and compacting chromatin structures in living cells
Source: Natl Sci Rev. 2025 Oct 21;13(3):nwaf451. doi: 10.1093/nsr/nwaf451 (PMC12875114; doi:10.1093/nsr/nwaf451)
Supplement: nwaf451_Supplemental_Files [file nwaf451_supplemental_files.zip › Supplementary Figure legends.docx]

**Supplementary Figure legends**

**Supplemental figure 1. Optical-feedback stabilization of MINFLUX nanoscopy. (a**) Optical-feedback stabilization unit including an example wideﬁeld image of the scattered reﬂection signal of gold nanorods in the sample. (**b**) Sample displacements Δx,y,z from the target position as measured by the active stabilization. Statistics shows Mean$\pm$S.D.

**Supplemental figure 2. Targeted coordinate patterns in 3D iteration sequence for MINFLUX 3D imaging. (a)** Targeted coordinate patterns in sequential iterations. (**b)** Critical parameters for 3D iteration sequence. (**c)** The acquisition time for single location of the fluorescent molecular, 5-HMSiR-Hoechst. (**d)** Distribution of acquisition time for locations of fluorescent dyes with MINFLUX 3D imaging. 5-HMSiR-Hoechst, DNA probe; Tom20-AF647, mitochondrial out-membrane protein, Tom, labeled with primary antibody conjugated with Alexa Fluor 647. **(e)** Distribution of acquisition time for locations of 5-HMSiR-Hoechst with MINFLUX 2D imaging. **(f)** One example of the imaging time of the chromatin fiber visualized in a large image within a living cell. This fiber-like chromatin structure is obtained within 3 seconds. Each location is color-coded by its time of the imaging process.

**Supplemental figure 3. MINFLUX precision for Tom20-AF647and DNA marker, 5-HMSiR-Hoechest. (a)**3D MINFLUX detection of Tom20-AF647 fluorophore in neuron. Tom20-AF647, mitochondrial out-membrane protein, Tom, labeled with primary antibody conjugated with Alexa Fluor 647.Parameters for detected single cluster are given. **(b)** 3D MINFLUX detection of 5-HMSiR-Hoechst in fixed cell. Peak counts in a single location higher than 3 are included. Parameters for the detected signal are given.

**Supplemental Figure 4. MINFLUX imaging of DNA probe 5-HMSiR-Hoechst in living cells. (a)** Confocal image and two-dimensional MINFLUX imaging of DNA stained with 5-HMSiR-Hoechst in living cell. Magenta (aggregated) and green (monomer) show JC-1, staining for functional mitochondrial in living cell. Red show 5-HMSiR-Hoechst, staining for DNA. **(b)** 2D MINFLUX Image shows spontaneous “blinking” event of 5-HMSiR-Hoechst. MINFLUX locations were acquired in the same cell every 3 minutes. Locations were color-coded according to the count number (upper panels) and localizing time (lower panels). Distinct DNA patterns in the same cell were observed at different time point. Locations in adjacent structures were localizing in a short time window.

**Supplemental figure 5. MINFLUX imaging of DNA fiber-like structures in living cells. (a,b)** Examples show the fiber-like structures of 3D MINFLUX localizing of DNA probes in living cells. Locations were color-coded with local density function. **(c)** Diameters of the fiber-like structures in living cells.

**Supplemental Figure 6. MINFLUX imaging of DNA fiber-like structures in fixed cells. (a)** Example shows the fiber-like structures detected by 3D MINFLUX localizing of DNA probes in the middle of a nucleus. small inserts (a-d) show transections at four locations of the chromatin fiber. **(b)** Example of the same area as (**a**), locations are color-coded with density function. Right, segments in the chromatin fiber and estimated DNA density in each segment. Estimation is done by counting probe numbers in the 3D volume of each segment. Each probe binds to 12 bp AT-rich DNA, and the AT-rich region in the chromatin fiber is estimated as 80% of DNA. **(c)** Examples of the fiber-like structures of 3D MINFLUX localizing of DNA probes in PFA-fixed cells. Estimated absolute DNA density in each fiber (Mbp/μm^3^). It is noted that due to the variation of AT-rich DNAs in each fiber, some fibers in the low end might be underestimated around 2-3-fold. **(d)** Another example of the fiber- like structures of 3D MINFLUX localizing of DNA probes in PFA-fixed cell. Small inserts show transections at three locations of the chromatin fiber. Loop-like DNA chain-like structures (lines) outside the fiber are detected in this case. Right, another two representative examples show the fiber-like structures of 3D MINFLUX localizing of DNA probes with variable widths in PFA-fixed cell. Locations are color-coded with density function.

**Supplemental Figure 7. Chromatin fiber detection in 2 color-3D MINFLUX. (a)** Confocal imaging and MINFLUX locations detected in dual-color -3D MINFLUX. DNA is probed by 5-HMSiR-Hoechest, histone is probed by H2A-CF680, H2A labeled with primary antibody conjugated with CF680. **(b)**Spectrum ratio of each detected location in (**a**). Two channels cover spectral fractions of 650nm-685 nm and 685-760 nm. **(c)** Example showed the dividing of each location based on the spectrum ratio. Spectrum distribution of each experiment is fitted by two Gaussian distribution function and the intersection of the two curves is used as the dividing point. Right, example of the MINFLUX locations assigned to the two probes. **(d)** The dividing points of spectrum ratio in each of the dual-color -3D MINFLUX experiment (DNA-H2A, DNA-H3K9me3) showed similar ratio intersection. The same fluorophores, 5-HMSiR and CF680, were used in the two sets of experiments. **(e)** Chromatin fiber-like ultrastructure visualized together with H2A-CF680 by dual-color -3D MINFLUX imaging. **(f)** Examples of chromatin fibers detected in dual-color -3D MINFLUX. DNA is probed by 5-HMSiR-Hoechest (red), H3K9me3 is probed by CF680 labeled antibody(green). Red signals are isolated and estimated diameters of each segment in the chromatin fiber (DNA-signal) are comparable to the chromatin fibers detected in one-color 3D MINFLUX.

**Supplemental figure 8. MINFLUX imaging of DNA fiber-like structures in cells.** Comparing of chromatin fiber parameters from samples with PFA fixation (N=26 cells) or Methanol fixation (N=31 cells).

**Supplemental figure 9. MINFLUX imaging of DNA fibers in primary culture of cortical neurons.** Statistical analysis of chromatin fiber parameters from samples with PFA fixation (N= 389 fibers from cells).

**Supplemental Figure 10. Ring-like structure of 3D MINFLUX localizing of probes in living cells. (a)** Four examples show the ring-like structures of 3D MINFLUX localizing of DNA probes. Locations in 3D-space were fitted with ring. D, diameter. **(b)** Quantifications of diameters of observed nucleosomes which are fitted by a cycle model. Two peaks indicate different orientation of the simulated plate. N=996.

**Supplemental Figure 11.** **MINFLUX imaging of nucleosomes in living cells.** Two examples show the nucleosome-like structures of 3D MINFLUX localizing of DNA probes in living cells. Locations were color-coded with sequence. Helical and Cylindrical model is used in the fitting. Illustration shows the mapping of probe locations of DNA probes on the nucleosome model (7Y5W).

**Supplemental Figure 12. MINFLUX imaging of nucleosome structures in fixed cells and living cell with cylindrical model fitting. (a, b**) Example of 3D MINFLUX localizations of 5-HMSiR-Hoechst in a fixed cell. Simulation shows the cylindrical model fitting of a nucleosome (each with > 5 DNA probe locations). **(c)** Examples of 3D MINFLUX localizations of 5-HMSiR-Hoechst in a living cell. Insert, cylinder fitting models of nucleosomes (each with > 5 DNA probe locations). Red, probe locations within nucleosome fitting models; Gray, probe locations without detectable nucleosome models. Statistics shows the diameter of the nucleosome structure, calculated as probe locations fitting with cylinder model of nucleosome.

**Supplemental Figure 13. MINFLUX imaging of dimmer-like nucleosomes in living cell. (a**) Example of 3D MINFLUX localizations of 5-HMSiR-Hoechst in 24x27x33 nm space. Simulation shows the helical & cylindrical model fitting of a nucleosome dimmer. (**b**）Two examples of 3D MINFLUX localizations of 5-HMSiR-Hoechst in 600x 500 x 450 nm space and 300x 240 x 180 nm space. Insert, cylinder fitting models of nucleosome dimmers. Magenta, probe locations within nucleosome fitting models; gray, probe locations without detectable nucleosome models. Statistics shows the diameter of the nucleosome dimmer structure, calculated as probe locations fitting with cylinder model of nucleosome dimmer (N=1329).

**Supplemental figure 14. Example of nucleosome units fitted by a helical & cylindrical model in the chromatin fiber. (a**) Examples of possible nucleosome tetramers in the chromatin fiber. Left, Example shows the Locations of DNA probes in the chromatin fiber regions by 3D-MINFLUX. Right, inserts show sub-regions in the chromatin fiber (i, ii) and their fitting result with the helical & cylindrical model.

S**upplemental figure 15. Example of nucleosome units fitted by a cylindrical model in the chromatin fiber. (a**) Examples show the Locations of DNA probes in the chromatin fiber regions by 3D-MINFLUX in PFA-fixed cell. (**b**) Inserts show sub-regions in the chromatin fiber(i,ii) and their fitting result with the cylindrical model. Example of mapping of probes suggest a possible nucleosome polymer (12-mer) in the fiber.

**Supplemental figure 16.** **MINFLUX imaging of DNA probe** **5-HMSiR-Hoechst in reconstituted chromatin. (a**) EM image and 2D MINFLUX image of the 12$\times$177-bp nucleosome arrays. Insert (i) shows the FWHM for the detected spot. (**b**) 3D-MINFLUX image of the 12X177-bp nucleosome array. Insert (*) shows probe locations fitted by nucleosome models. **(c)** EM image and 3D-MINFLUX image of the reconstituted chromatin fiber (12$\times$177-bp). Examples show the 30nm-chromatin fibers.

**Supplemental figure 17. TSA-induced alternation of nucleosome compaction in living cells.**

**(a**) Three-dimensional MINFLUX imaging of DNA stained with 5-HMSiR-Hoechst in living cell. Two examples were shown. Gray, probe locations without detectable nucleosome models; Red, probe locations fitted by nucleosome models (>5 points for each nucleosome). **(b)** Cells are treated in TSA for 12 hours. Examples show the probe density and nucleosome model fitting data in the same cell. Insert, probe locations fitted by a nucleosome model.
