# Supplementary material for: MINFLUX dissects nucleosome and compacting chromatin structures in living cells
Source: Natl Sci Rev. 2025 Oct 21;13(3):nwaf451. doi: 10.1093/nsr/nwaf451 (PMC12875114; doi:10.1093/nsr/nwaf451)

Supplementay Figures

Supplemental figure 1.

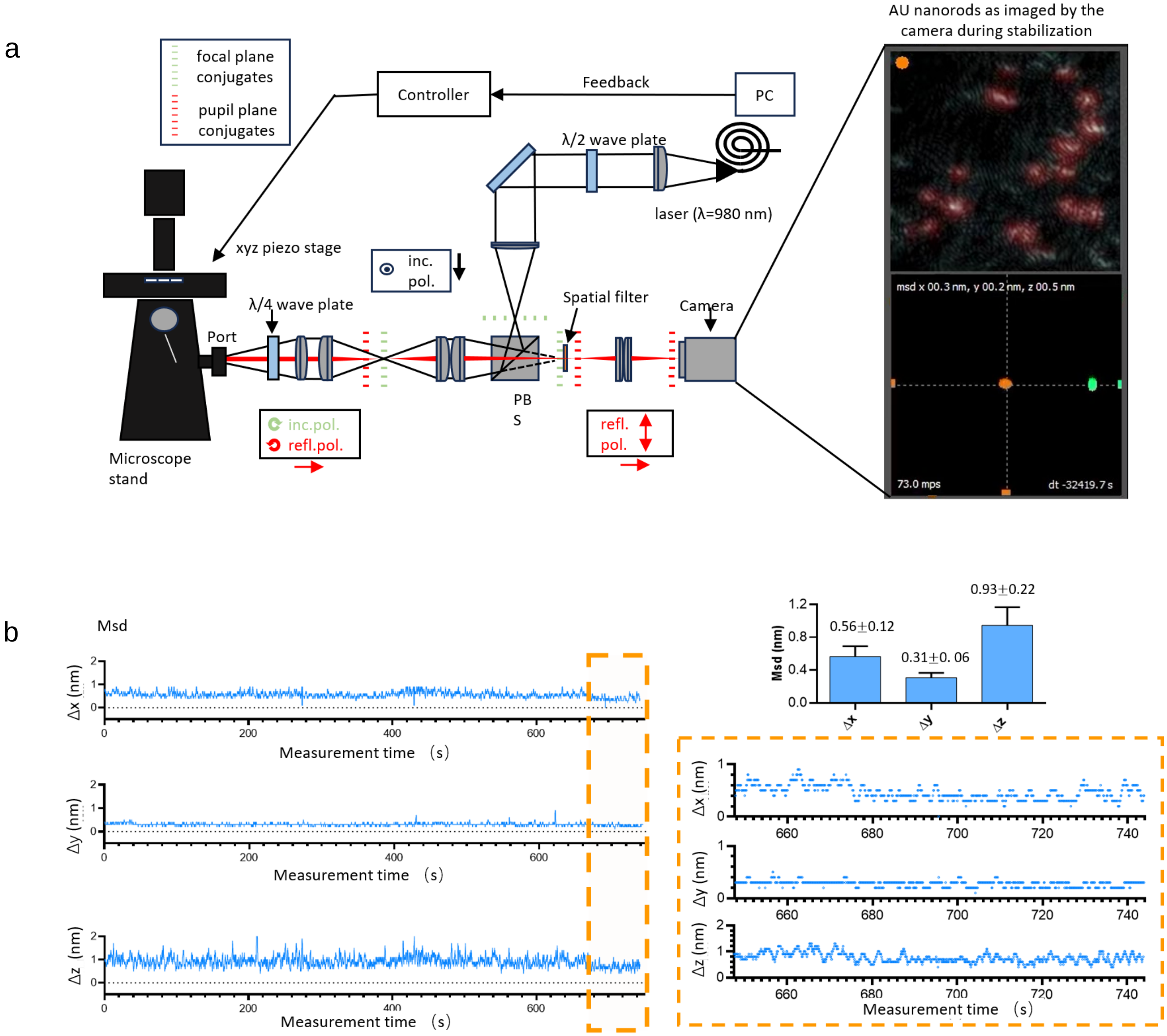

Supplemental figure 2.

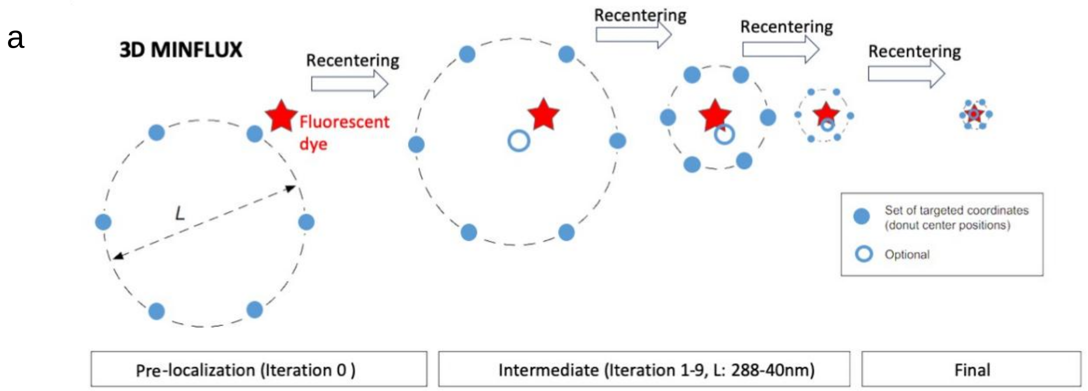

b

Critical parameters for 3D iteration sequence

Iteration sequency	0	1	2	3	4	5	6	7	8	9
L (nm)	Pre-localization	288	288	288	151	151	76	76	40	40
CFR	-3.05E-05	-3.05E-05	-3.05E-05	-3.05E-05	-3.05E-05	-3.05E-05	-3.05E-05	-3.05E-05	-3.05E-05	-3.05E-05
Laser power factor	1	1	1	1	2	2	4	4	6	6
CCR limit	off	off	off	off	0.9	off	0.8	off	off	off
Minimal photon count	282	439	260	253	525	422	787	788	1038	1082
EFO (kHz)	49.2	130.7	88	91	82	91	146	128	173	172

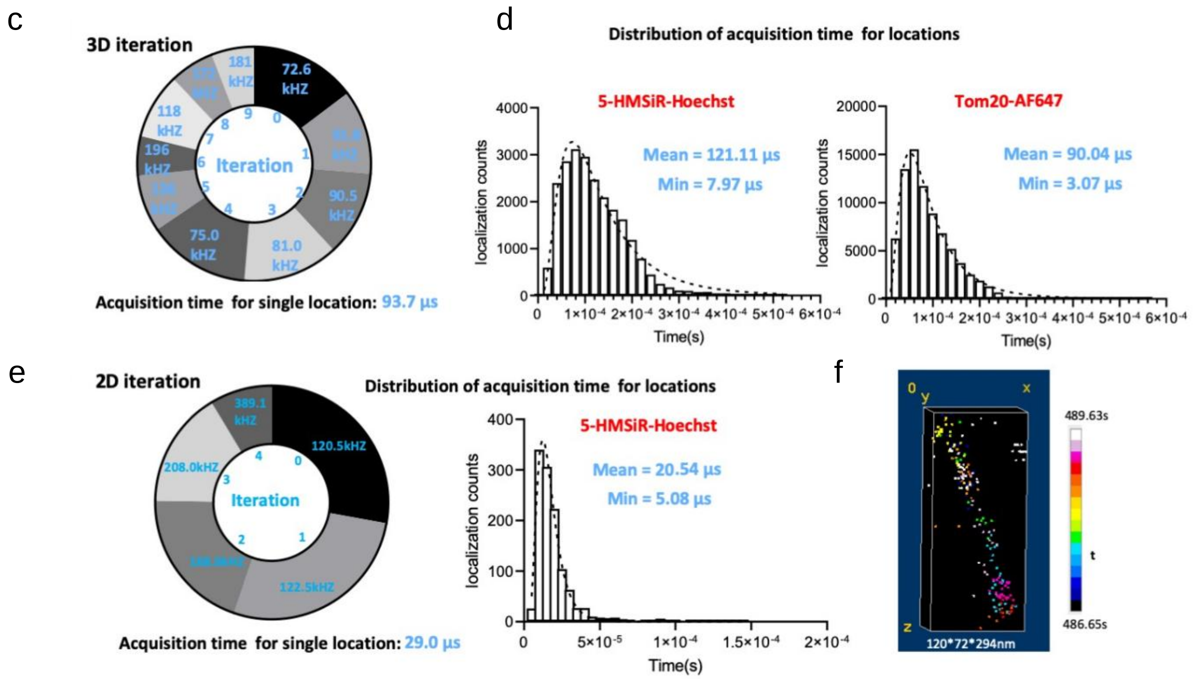

Supplemental figure 3.

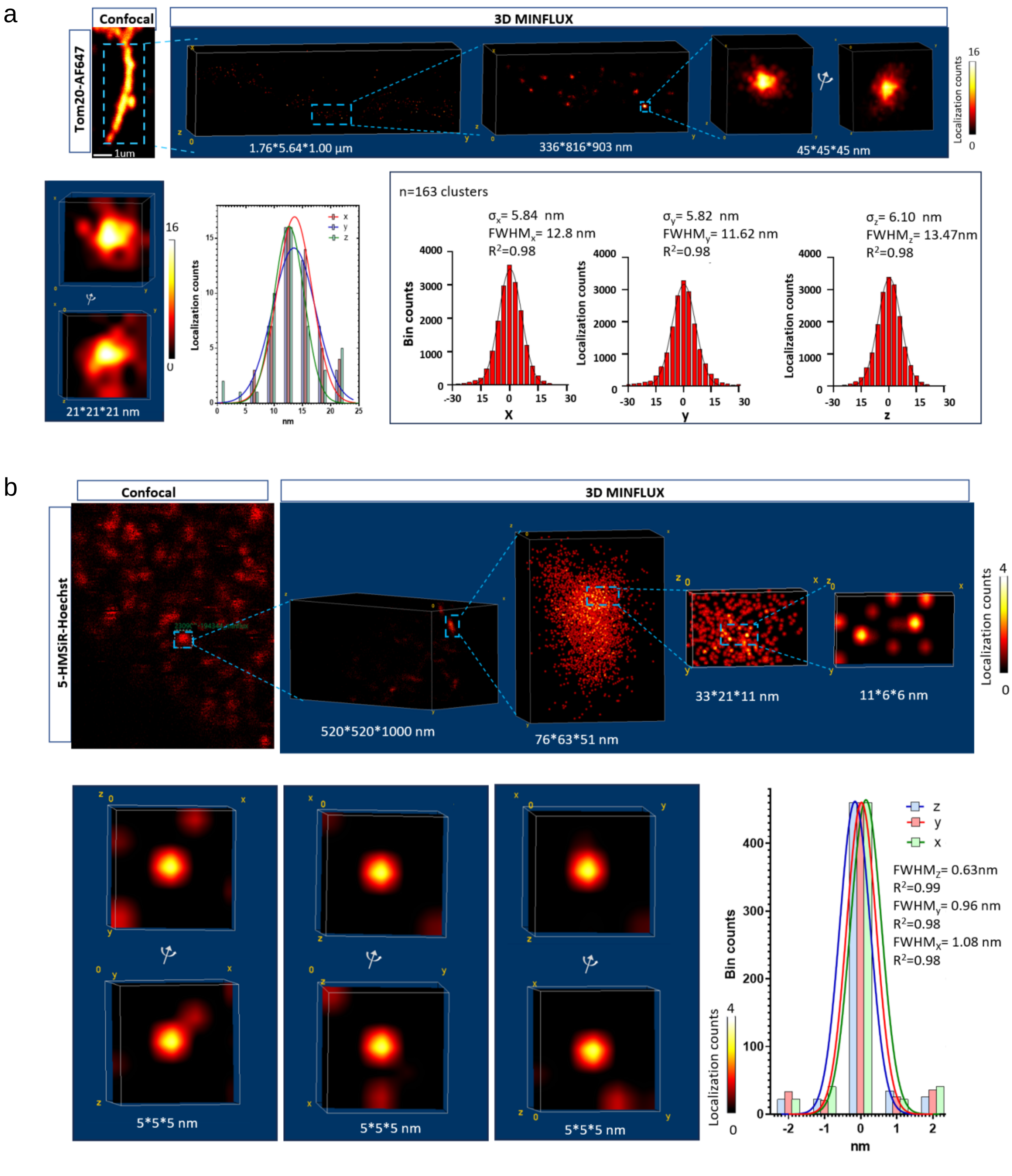

Supplemental figure 4.

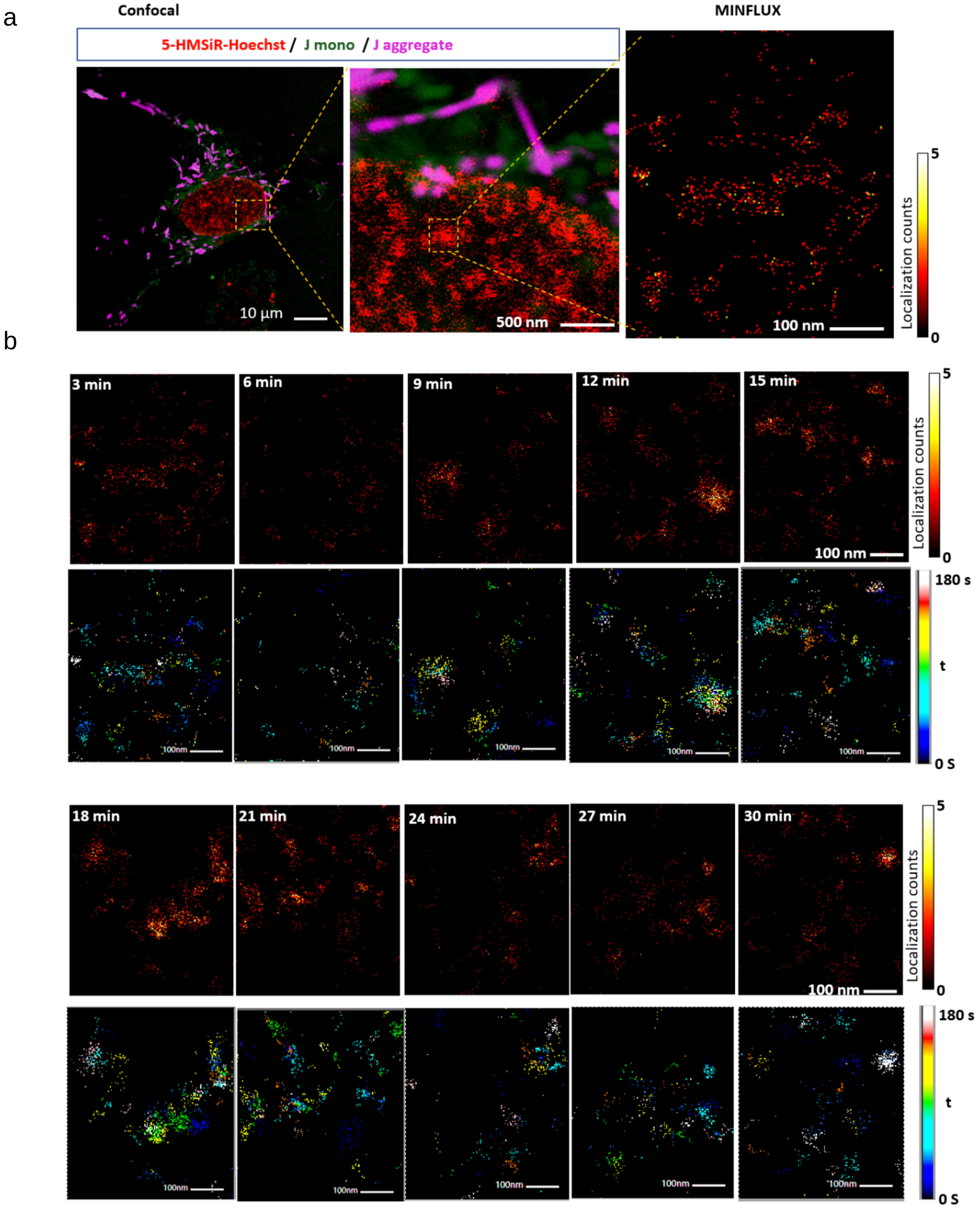

Supplemental Figure 5.

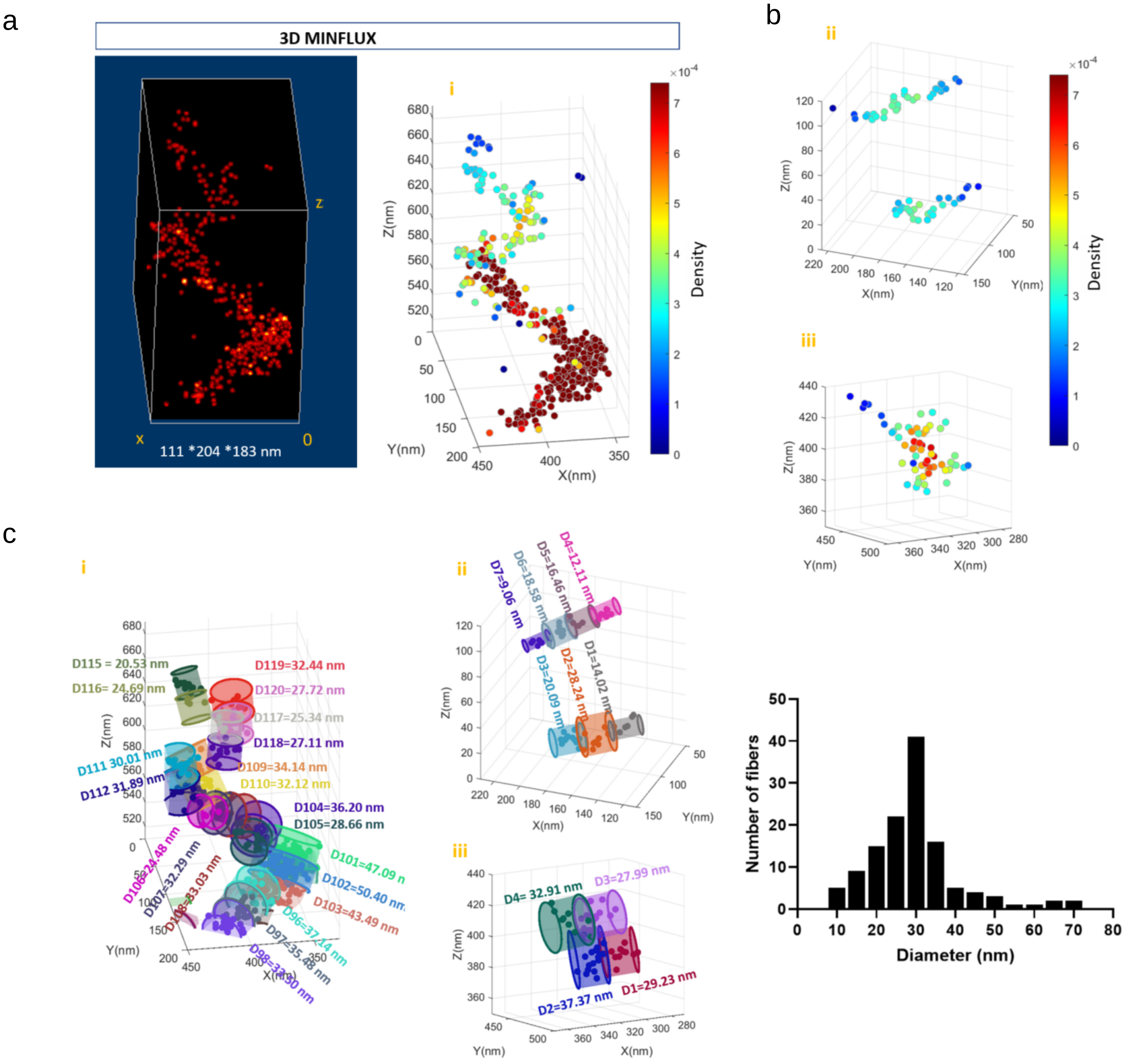

Supplemental figure 6.

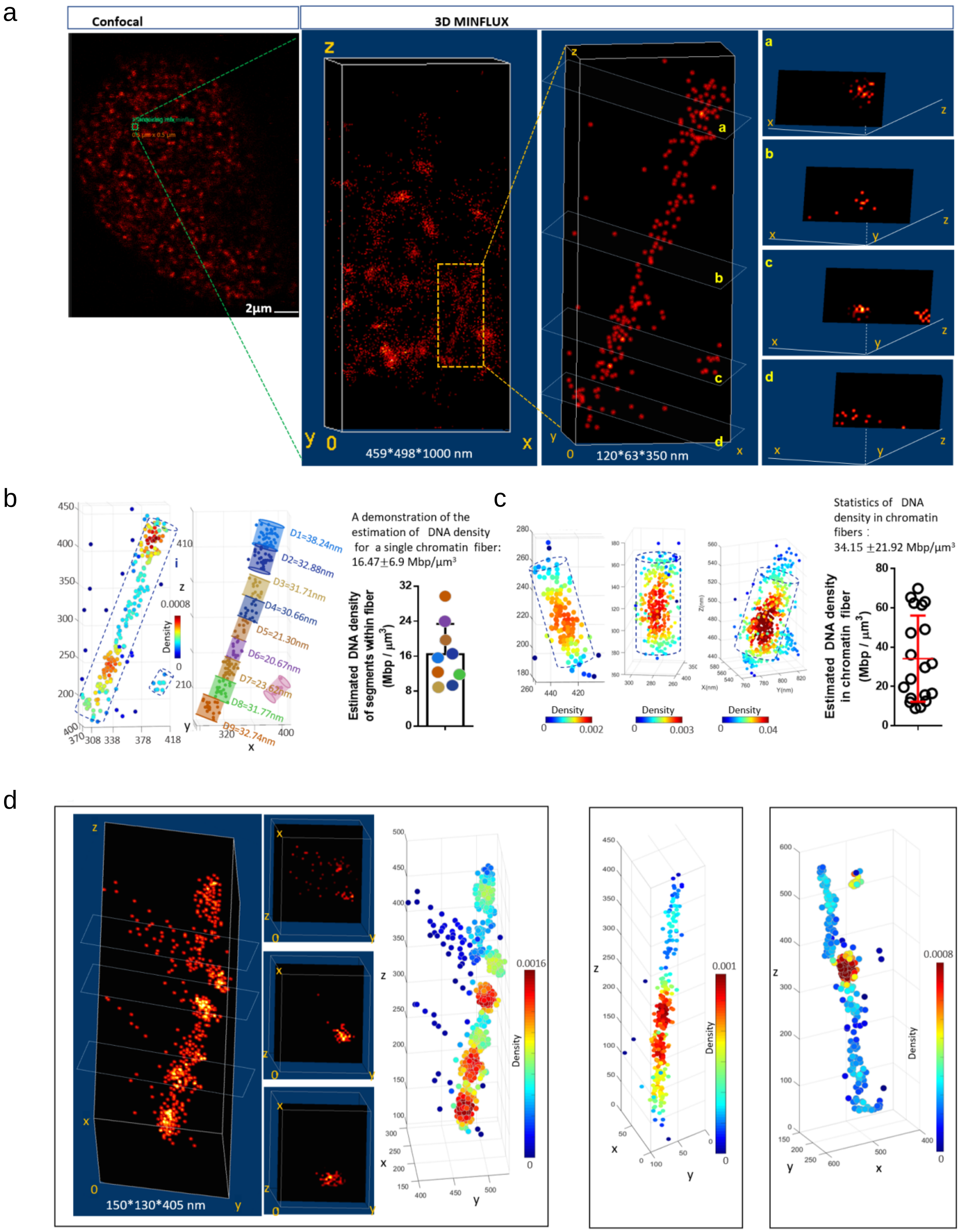

Supplemental Figure 7.

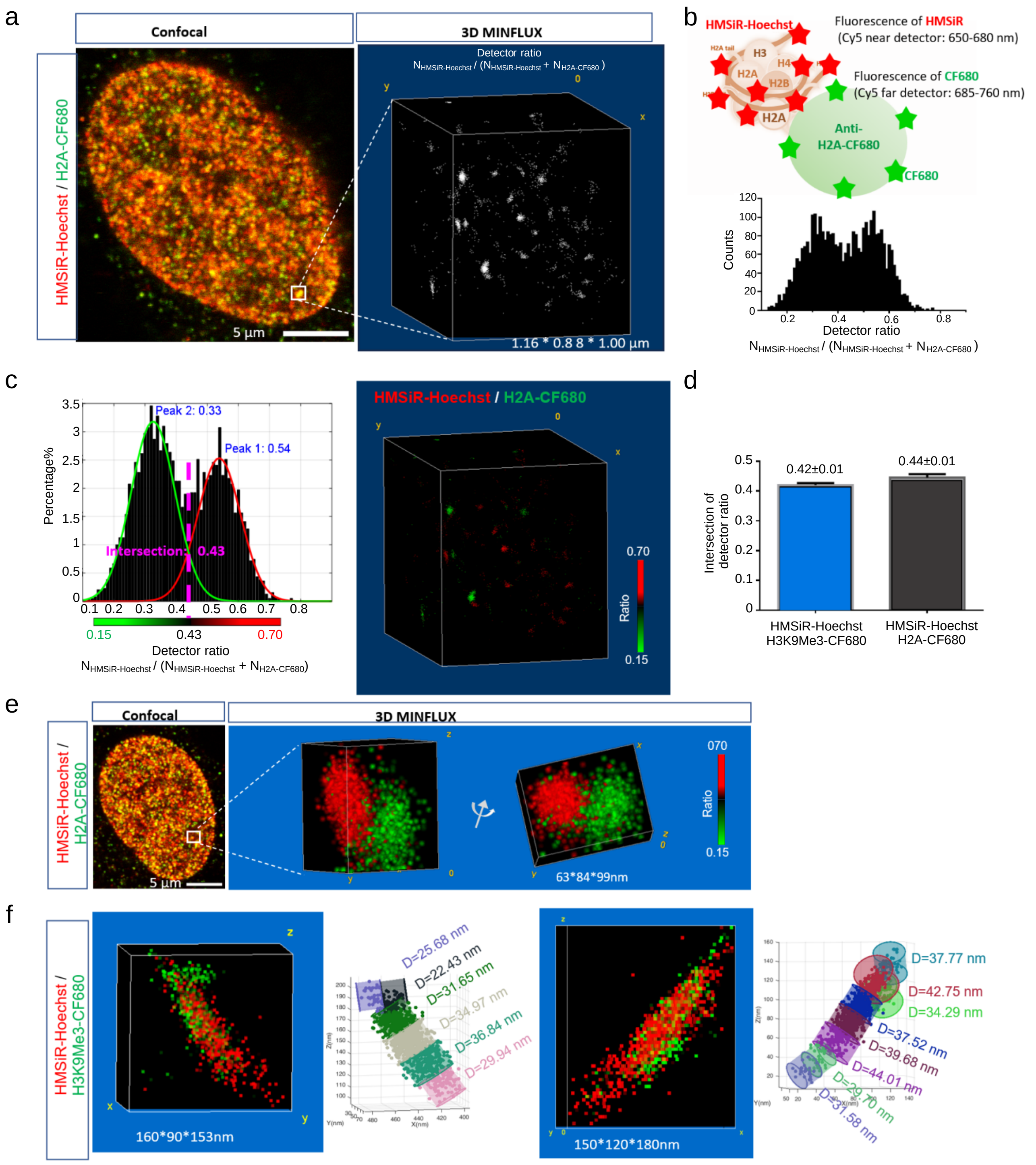

Supplemental Figure 8.

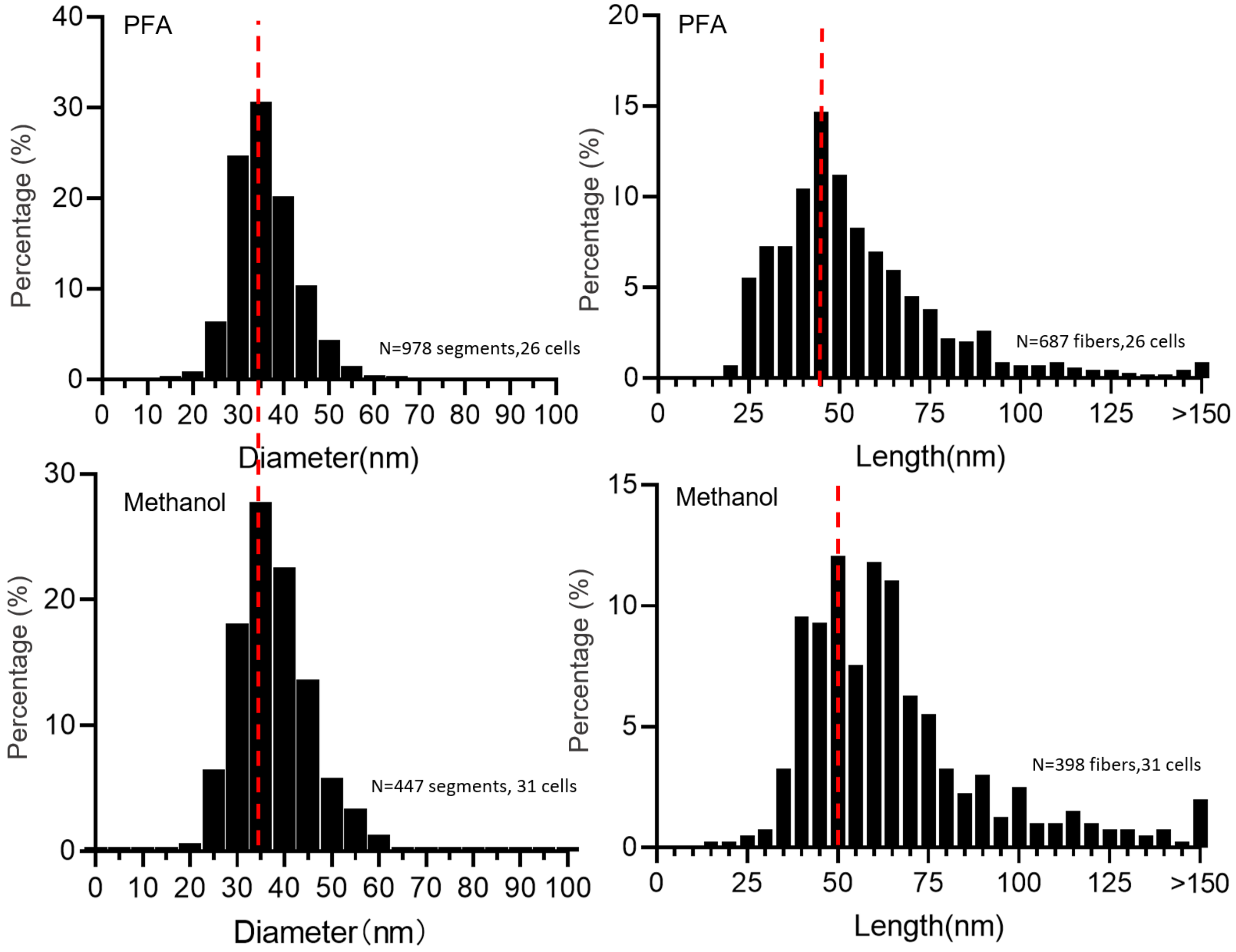

Supplemental figure 9.

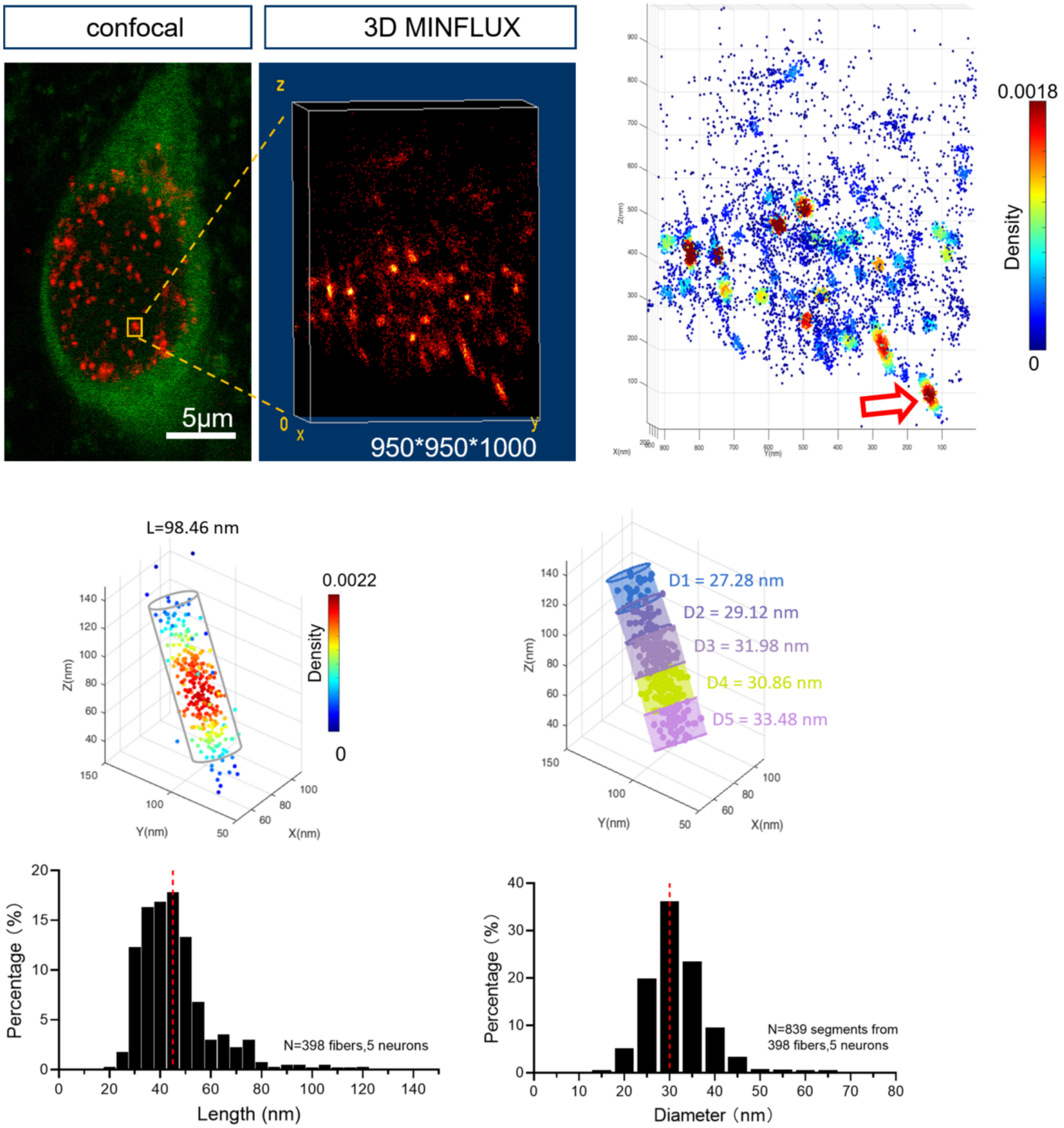

Supplemental figure 10.

a

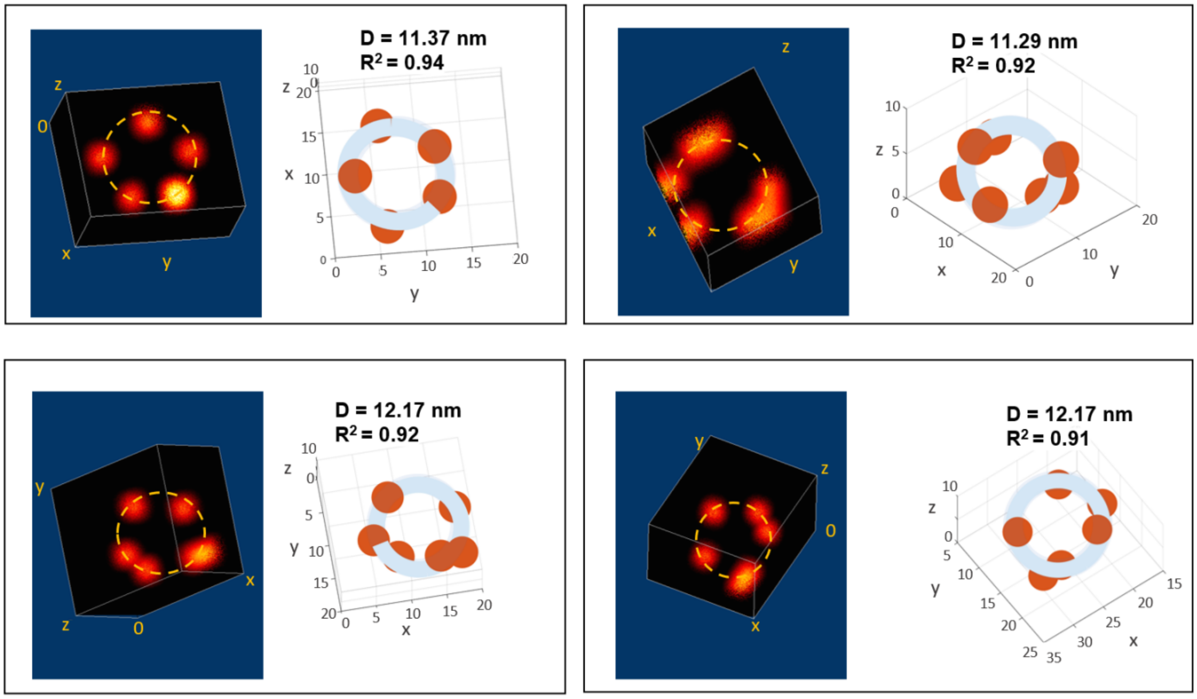

b

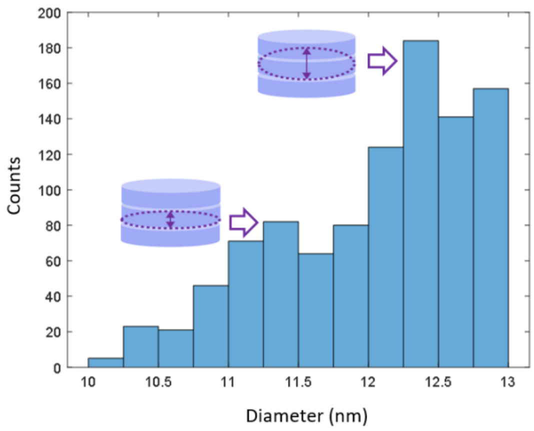

Supplemental Figure 11.

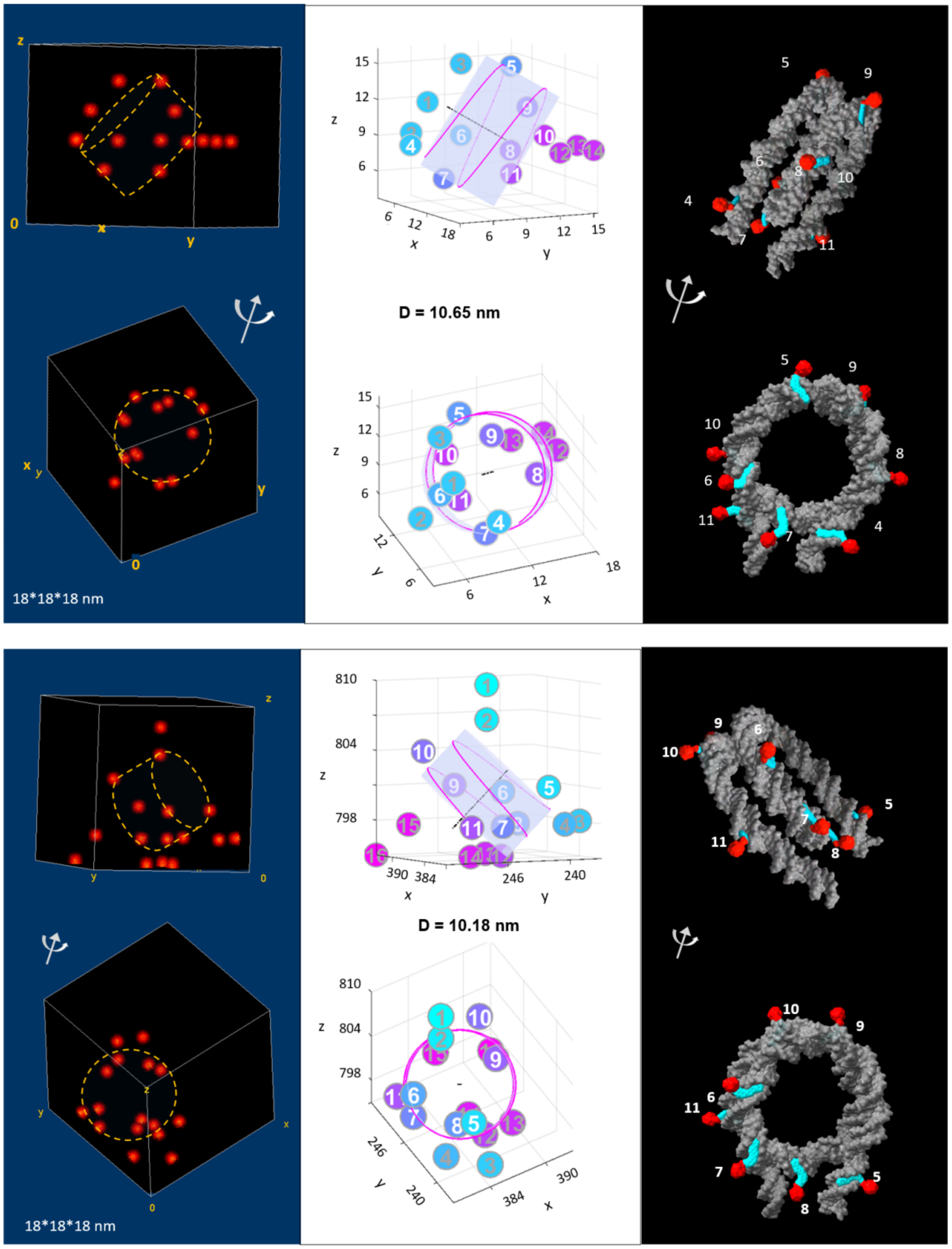

Supplemental Figure 12.

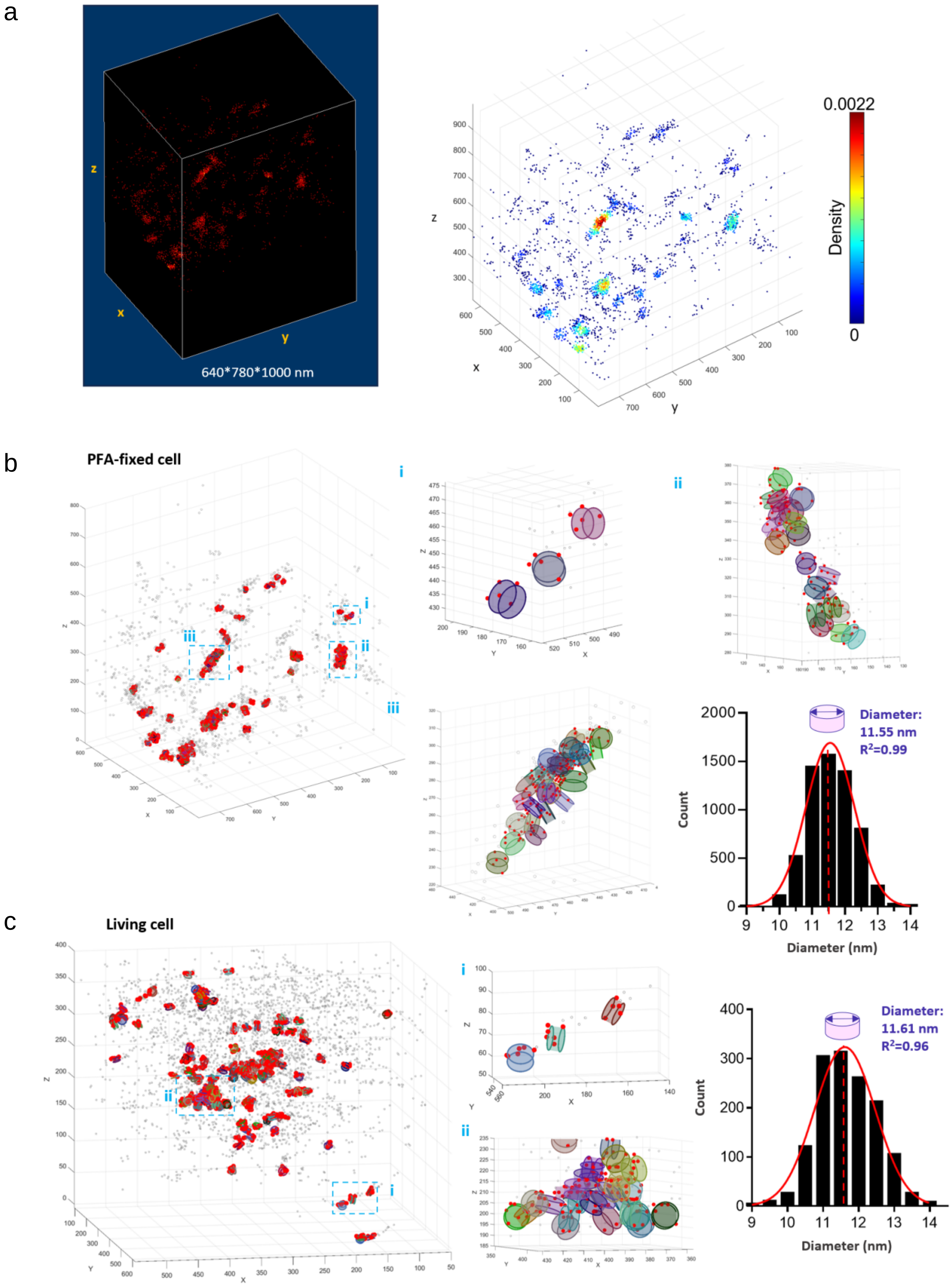

Supplemental Figure 13.

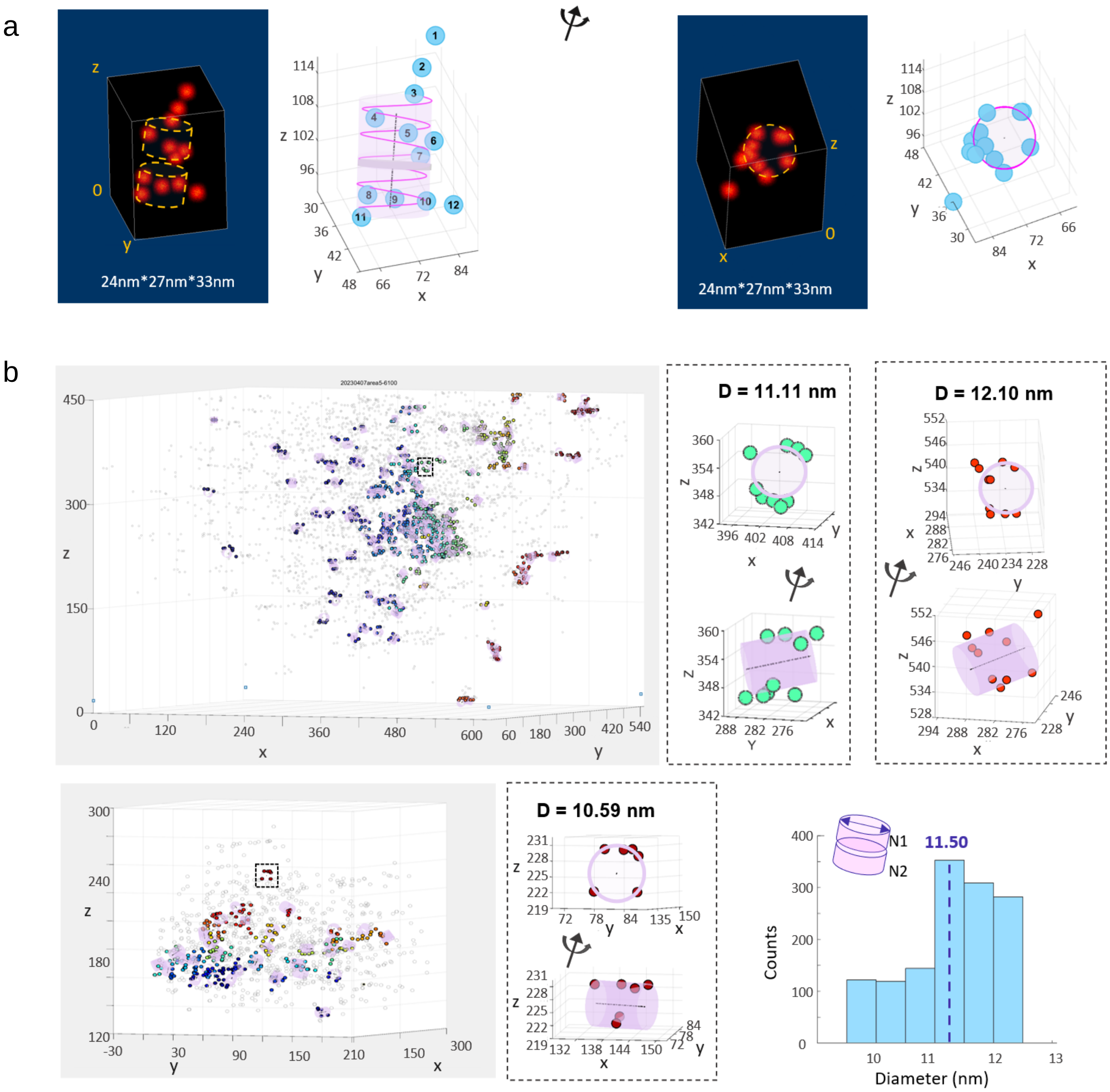

Supplemental Figure 14.

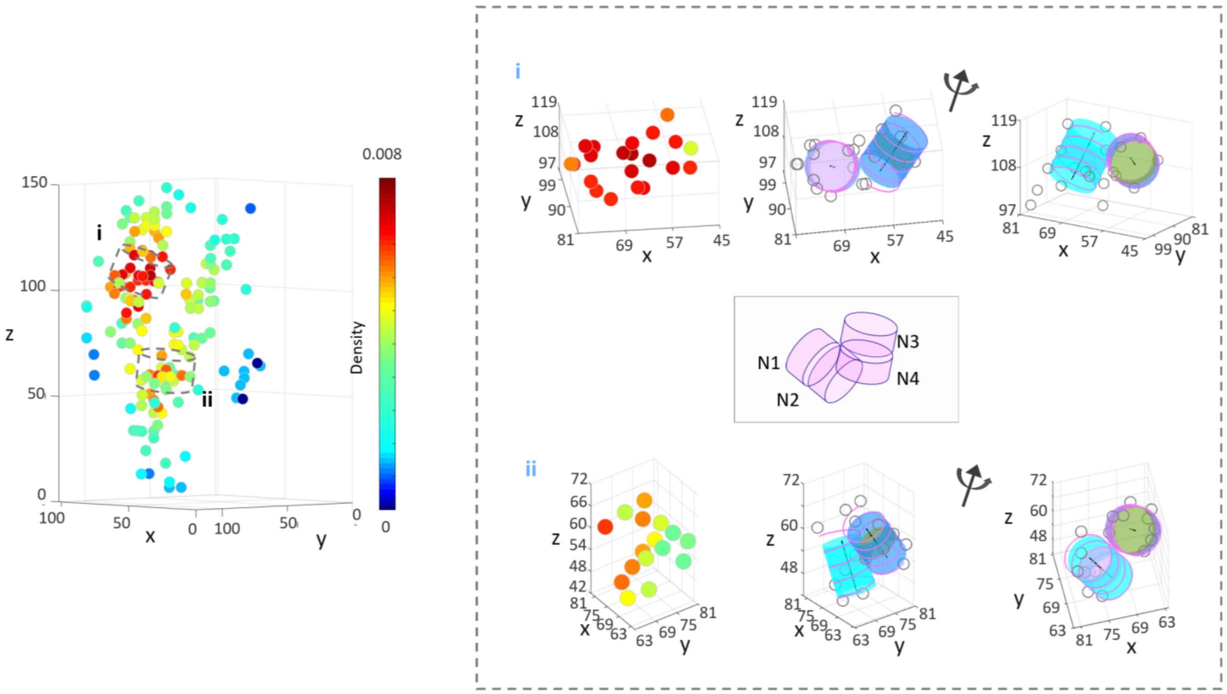

Supplemental figure 15.

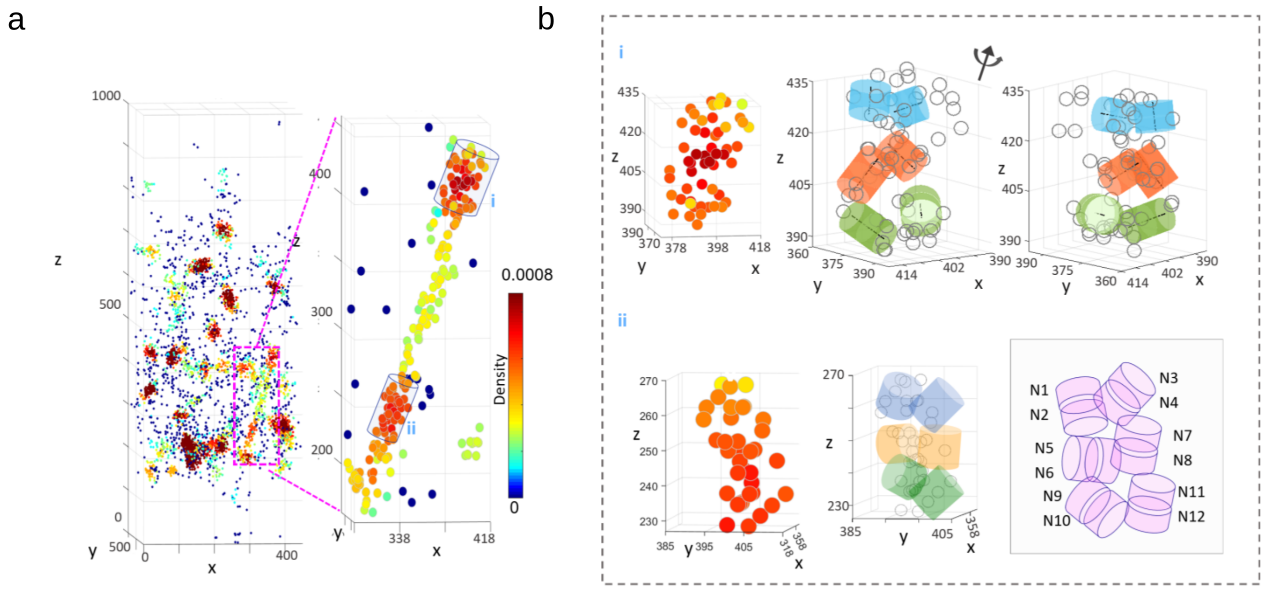

Supplemental figure 16.

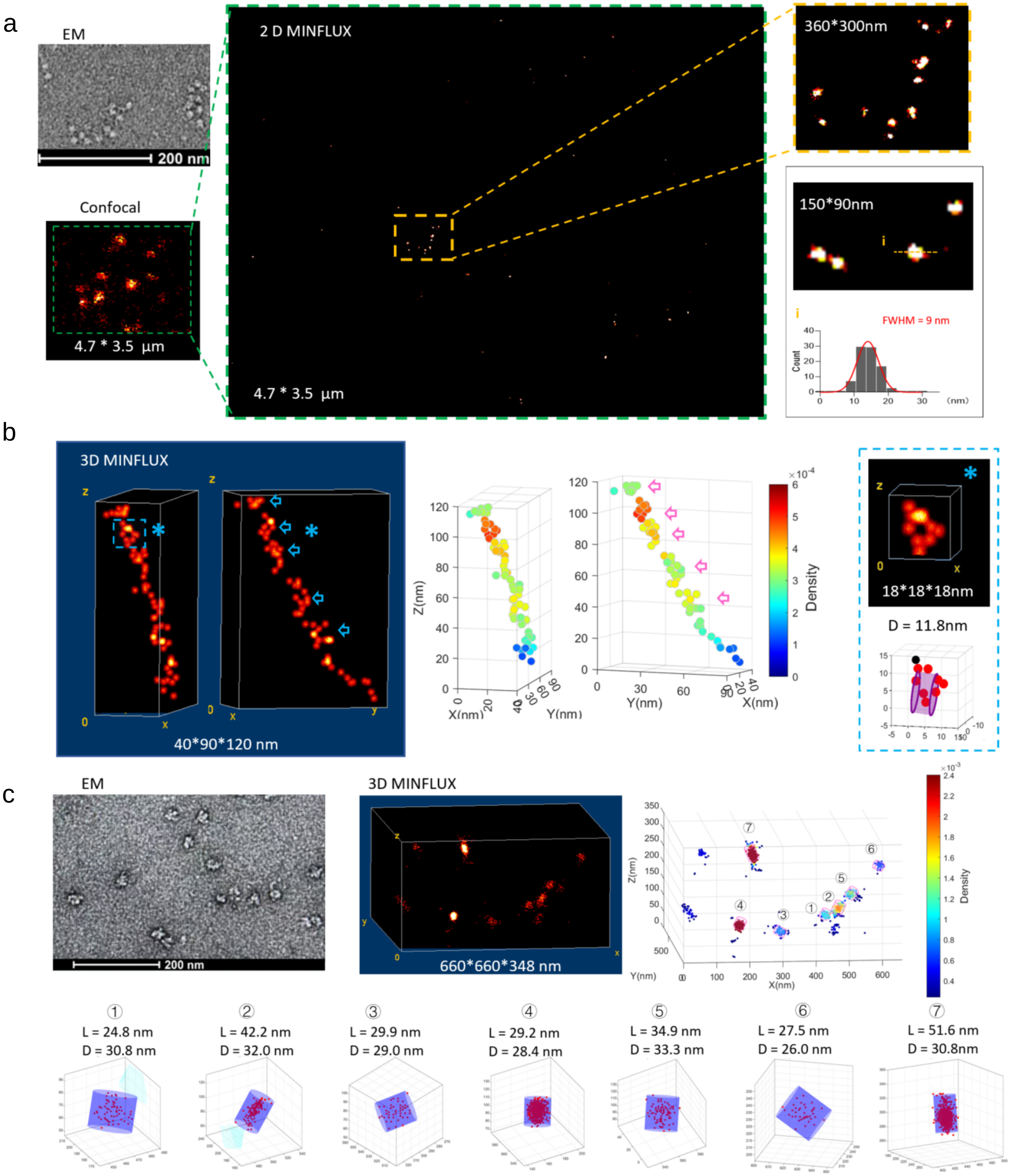

Supplemental figure 17

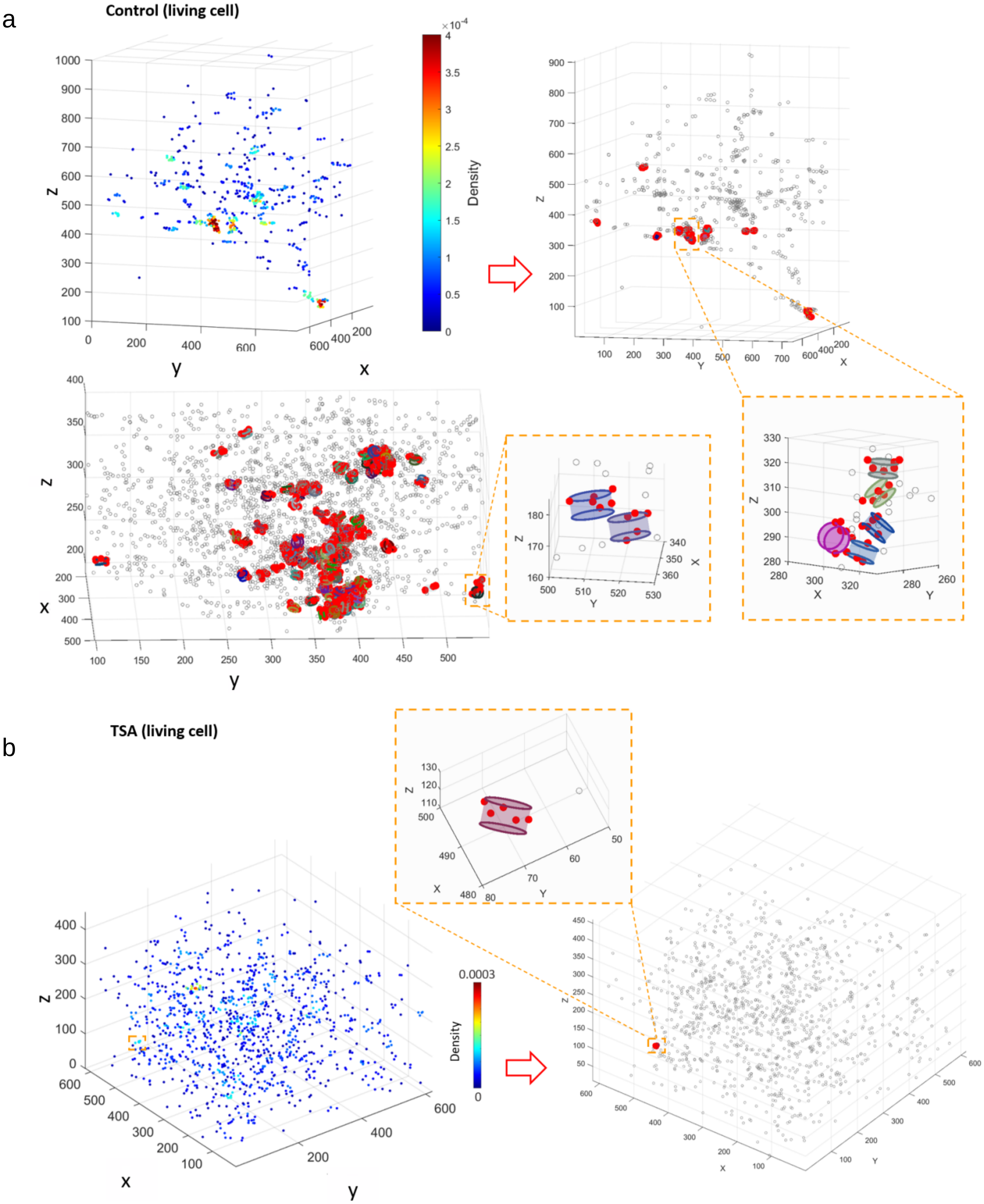

Supplement: nwaf451_Supplemental_Files [file nwaf451_supplemental_files.zip › Supplementary Figures- 2.pdf]
