# Supplementary material for: MINFLUX dissects nucleosome and compacting chromatin structures in living cells
Source: Natl Sci Rev. 2025 Oct 21;13(3):nwaf451. doi: 10.1093/nsr/nwaf451 (PMC12875114; doi:10.1093/nsr/nwaf451)
Supplement: nwaf451_Supplemental_Files [file nwaf451_supplemental_files.zip › Supplementary Materials and Methods-1.docx]

**Supplementary Materials and Methods**

**Maintenance and preparation of cell culture**

U2OS cells (human osteosarcoma cell line) were cultured in Dulbecco's Modified Eagle's Medium (DMEM, Gibco), supplemented with 10% fetal bovine serum (FBS, Gibco), and 1% penicillin-streptomycin. The cells were maintained at 37°C with 5% CO2 in an incubator (Thermo). When U2OS cells reached 70-80% confluence, they were subcultured. The culture medium was aspirated, and cells were washed with phosphate-buffered saline (PBS, Gibco). A 0.5% trypsin solution (Sigma Aldrich) was added for 2 minutes to detach cells, which were then neutralized with fresh DMEM containing FBS. The cell suspension was transferred to a new culture vessel with fresh medium. Cell cultures were regularly inspected for contamination and monitored for cell morphology, growth rate, and confluence. Primary cultures of neurons were prepared as previous report[1]. Cortical cultures were prepared from embryonic day 16 (E16) ICR mice (4–5 mice per culture experiment), with 0.125% trypsin in the presence of 0.05% DNase I (Sigma). Cells (5 × 10^5^ per cm^2^) were maintained in Neurobasal medium (Invitrogen) containing 2% B27 supplement, 2 mM Glutamax and penicillin/streptomycin (Invitrogen) for 12–14 d.

**Live cell status monitoring using JC-1 staining**

To assess cellular status during live-cell imaging, we utilized the mitochondrial membrane potential dye JC-1. While before conducting MINFLUX DNA imaging, mitochondrial imaging was performed using a confocal module. Specifically, following the completion of DNA probe staining, we applied the commercially available JC-1 assay kit (Invitrogen). The JC-1 dye from the assay kit was added to the culture dish along with the DNA probe. Subsequently, the culture dish was returned to the incubator. During imaging, a 488 nm excitation laser was employed, and emitted light was filtered at 530 nm and 590 nm, corresponding to the peak fluorescence from the monomer and aggregate signals, respectively[2, 3].

**DNA Staining and cell fixing with 5-HMSiR-Hoechst Probe**

For fixed cell experiments, U2OS cells were allowed to grow on cover glasses (Deckgläser, diameter: 18mm) for 12 hours. Before staining, the cell samples were gently washed with pre-warmed PBS. Subsequently, fresh maintenance medium was added, and the staining solution containing 5-HMSiR-Hoechst with a final concentration of 2nM was introduced. The dish was then returned to the CO2 incubator for 2 hours. Fixation was carried out using two different methods: -20°C pre-chilled 100% methanol, 4% PFA paraformaldehyde (PFA, Sigma Aldrich) at room temperature, all for a fixed duration of 10 minutes. Following fixation, U2OS cells were rinsed three times with PBS for five minutes each. After fixation, cover glasses were treated with a solution of gold nanoparticles and left to stand on ice for 30 minutes. Subsequently, the cells were washed three times with PBS. Cover glasses with U2OS cells were sealed using silicone rubber (Zhermack) on glass slides. All procedures following fixation were conducted under light-avoidance conditions.

**DNA Staining with 5-HMSiR-Hoechst Probe in Reconstituted Chromatin​**

Glass coverslips (Deckgläser, 18 mm diameter) were pre-treated with poly-D-lysine (PDL, Sigma-Aldrich) overnight, washed three times with sterile ddH₂O, and subsequently incubated for 1 hour with 150 nm gold nanoparticle solution (BBI Solutions). After each treatment step, coverslips were thoroughly rinsed with ddH₂O to remove residual reagents. Nucleosomal arrays and 30-nm chromatin fibers were reconstituted using a 12-mer 177-bp repeat 601 based DNA template via salt gradient dialysis with recombinant histone octamers, following established protocols [4]. The chromatin sample was diluted to a final concentration of 5-10 ng/μL in HE buffer (10 mM HEPES, pH 8.0, 0.1 mM EDTA), and 5-HMSiR-Hoechst was added to a final concentration of 12.5 pM. The 60 μL staining mixture was evenly applied to pre-treated coverslips and incubated at 4 °C for 2 hours. After incubation, the staining solution was gently removed, and samples were mounted with fresh HE buffer for MINFLUX imaging.

**Dual-Color Staining of DNA and Histone Markers for MINFLUX Imaging**

For the dual-color staining protocol, the initial staining with 5-HMSiR-Hoechst and cell fixation were performed following previously established protocols. Briefly, after fixation with 4%PFA, cells were washed three times with PBS, each wash lasting 5 minutes. To facilitate antibody penetration, cells were permeabilized with 0.3% Triton X-100 for 5 minutes, followed by blocking with 5% bovine serum albumin (BSA) for 25 minutes to prevent non-specific binding. Both permeabilization and blocking steps were conducted at 4°C.Antibodies against H3K9Me3 (ab184677) and H2A (ab177308) were conjugated with CF680 dye using the Mix-n-Stain™ CF™ 680 Antibody Labeling Kit (Biotium) according to the manufacturer's instructions. The conjugated antibodies were then diluted to 1:1000 for H3K9Me3-CF680 and 1:200 for H2A-CF680, respectively. A volume of 60 µL of each diluted antibody solution was carefully applied onto the blocked cover glasses and incubated at room temperature for 1 hour in a humidified chamber to achieve optimal binding. Following the primary antibody incubation, cells were washed three times with PBS to remove unbound antibodies. Post-washing, cells were incubated in GLOX buffer (pH 7.1) . The GLOX buffer was freshly prepared and contained 50 mM TRIS/HCl, 10 mM NaCl, 10% (w/v) glucose, 64 µg/mL catalase, 0.4 mg/mL glucose oxidase, and 3 mM mercaptoethylamine (MEA). Finally, cover glasses were sealed onto glass slides with silicone rubber (Zhermack) to minimize oxygen exposure and prevent sample dehydration during imaging.

**MINFLUX image rendering in three dimensions**. ImageJ was employed for processing and visualization of these grayscale images, enabling qualitative visualization of the data. Each localization event was substituted with a Gaussian distribution and visually depicted with chromatic coding based on the event count value. Images were systematically rotated in the built-in 3D viewer to capture snapshots from diverse orientations and subsequently amalgamated into an animation. Then, the MINFLUX dataset was imported into MATLAB, where localization clusters corresponding to distinct events were marked with distinct colors. The targeted regions of interest were then isolated and represented using MATLAB's 3D scatter plot function, facilitating comprehensive exploration and analysis.

**MINFLUX Localization and Structural Analysis**

**Data Extraction and Pre-processing:** Raw MINFLUX imaging data, containing the locations of individual fluorescent molecules, were processed using a custom MATLAB code. Initial analyses focused on nucleosome and dimeric structure investigations without applying any filtering or averaging.

**Free Energy Map (density) Calculation:** DNA probe locations detected by the MINFLUX system were used to generate a normalized free energy map to evaluate the density in each probe location. Beginning with the determination of the total number of particles, the Euclidean distances between each particle and all others are systematically computed. The subsequent quantification of neighboring particles within a defined radius allows for the calculation of a normalized free energy map. This map reflects the local particle density around each DNA probe localization, with color-coded markers in a 3D scatter plot representing the computed free energy values.

**Chromatin Length and Diameter Measurement**: To assess the lengths and diameters of chromatin fibers, large chromatin fiber clusters were separated using Density-Based Spatial Clustering of Applications with Noise (DBSCAN2), as previously reported[5]. Each identified chromatin fiber cluster (condensed DNA signal) was assigned a unique numerical identifier. Unclustered points were deemed representative of individual nucleosomes or DNA loop structures and were excluded from subsequent analyses. For every chromatin cluster identified by DBSCAN2, a segmentation approach was employed. The previously mentioned cylindrical fitting method was applied to segmentally fit each section of the chromatin cluster, enabling the measurement of the diameter and height of each cylindrical segment. This step-by-step analysis allowed for the quantification of both length and diameter of individual chromatin fibers within the imaged samples. The length of each chromatin fiber is long-axis within the chromatin fiber cluster. The exclusion of unclustered points ensured that only larger chromatin structures were considered in the subsequent measurements, enhancing the specificity of the analysis. For detailed step-by-step information and algorithmic details, please refer to the comments and documentation within the provided MATLAB code files. Main function ‘*data_cluster_base_edge_main.m’*: clustering 3D data points and fit cylindrical structures and measure the length of each structure. ‘data_cut_Radii_main2.m’：make segments of each cylindrical structures based on the height of the tetranucleosomal structural model and measure the diameter of each segment.

**Helical and Cylindrical Structure Analysis:** For the analysis of helical structures, a parametric equation for a helix was employed to generate 1000 points. A rotation matrix was then defined based on the axis direction of the cylinder and its angle with the Z-axis. MINFLUX localizations were subsequently transformed onto the surface of a cylinder. Cylindrical Height Calculation: The height (h) of the cylinder was determined by fitting a sphere to the scattered points. The Mean Absolute Error (MAE) loss was constructed, where *y_i_* represents the distance of point *i* to the center (average of coordinates *xyz)*, and *y_hati_* is the radius of the sphere. The iterative method was employed to minimize the MAE loss, and the cylinder height was set as *h = 2 x radius_sphere_*. Cylindrical Radius Calculation: The cylindrical radius *r_cylinder_* was determined by constructing an MAE loss, where *y_i_* is the distance of point *i* to the axis line AB of the cylinder. An iterative method was used to minimize the MAE loss, resulting in the determination of *r_cylinder_.* Cylindrical Axis Determination: On the basis of sphere fitting, 900 points were selected on the sphere. An exhaustive search was conducted to find the two points on the sphere that minimized the variance of distances from all points to the axis line AB, thus defining the axis of the cylinder. The entire process of helical and cylindrical structure analysis was performed without applying any filtering or averaging to the MINFLUX localizations.

The MATLAB code used for data analysis of this study is available at the following URL: https://github.com/Xiehong-usst/DNA-code..git.

**Reference**

1. Ding X, Liu S, Tian M *et al.* Activity-induced histone modifications govern Neurexin-1 mRNA splicing and memory preservation. *Nat Neurosci*. 2017; **20**(5): 690–699. doi: 10.1038/nn.4536

2. Smiley ST, Reers M, Mottola-Hartshorn C *et al.* Intracellular heterogeneity in mitochondrial membrane potentials revealed by a J-aggregate-forming lipophilic cation JC-1. *Proc Natl Acad Sci U S A*. 1991; **88**(9): 3671–3675. doi: 10.1073/pnas.88.9.3671

3. Reers M, Smith TW, Chen LB. J-aggregate formation of a carbocyanine as a quantitative fluorescent indicator of membrane potential. *Biochemistry*. 1991; **30**(18): 4480–4486. doi: 10.1021/bi00232a015

4. Song F, Chen P, Sun D *et al.* Cryo-EM study of the chromatin fiber reveals a double helix twisted by tetranucleosomal units. *Science*. 2014; **344**(6182): 376–380. doi: 10.1126/science.1251413

5. Pape JK, Stephan T, Balzarotti F *et al.* Multicolor 3D MINFLUX nanoscopy of mitochondrial MICOS proteins. *Proc Natl Acad Sci U S A*. 2020; **117**(34): 20607–20614. doi: 10.1073/pnas.2009364117
